# The World Federation of Societies of Biological Psychiatry guidelines on the assessment and pharmacological treatment of compulsive sexual behaviour disorder

**DOI:** 10.1080/19585969.2022.2134739

**Published:** 2022-11-17

**Authors:** Daniel Turner, Peer Briken, Joshua Grubbs, Leo Malandain, Gemma Mestre-Bach, Marc N. Potenza, Florence Thibaut

**Affiliations:** a Department of Psychiatry and Psychotherapy, University Medical Center Mainz, Mainz, Germany; b Institute for Sex Research, Sexual Medicine, and Forensic Psychiatry, University Medical Center Hamburg-Eppendorf, Hamburg, Germany; c Department of Psychology, Bowling Green State University, Bowling Green, OH, USA; d Department of Psychiatry and Addictive Disorders, University Hospital Cochin (site Tarnier) AP-HP, Paris, France; e Facultad de Ciencias de la Salud, Universidad Internacional de La Rioja, La Rioja, Spain; f Departments of Psychiatry and Neuroscience and Child Study Center, Yale School of Medicine, New Haven, CT, USA; g Connecticut Mental Health Center, New Haven, CT, USA; h Connecticut Council on Problem Gambling, Wethersfield, CT, USA; i Wu Tsai Institute, Yale University, New Haven, CT, USA; j INSERM U1266, Institute of Psychiatry and Neurosciences, University of Paris Cité, Paris, France

**Keywords:** Compulsive sexual behaviour disorder, hypersexuality, addictive behaviours, compulsive behaviours, sexual addiction, paraphilias, chemsex

## Abstract

**Objectives:**

The current guidelines aim to evaluate the role of pharmacological agents in the treatment of patients with compulsive sexual behaviour disorder (CSBD). They are intended for use in clinical practice by clinicians who treat patients with CSBD.

**Methods:**

An extensive literature search was conducted using the English-language-literature indexed on PubMed and Google Scholar without time limit, supplemented by other sources, including published reviews.

**Results:**

Each treatment recommendation was evaluated with respect to the strength of evidence for its efficacy, safety, tolerability, and feasibility. Psychoeducation and psychotherapy are first-choice treatments and should always be conducted. The type of medication recommended depended mainly on the intensity of CSBD and comorbid sexual and psychiatric disorders. There are few randomised controlled trials. Although no medications carry formal indications for CSBD, selective-serotonin-reuptake-inhibitors and naltrexone currently constitute the most relevant pharmacological treatments for the treatment of CSBD. In cases of CSBD with comorbid paraphilic disorders, hormonal agents may be indicated, and one should refer to previously published guidelines on the treatment of adults with paraphilic disorders. Specific recommendations are also proposed in case of chemsex behaviour associated with CSBD.

**Conclusions:**

An algorithm is proposed with different levels of treatment for different categories of patients with CSBD.

## Introduction

The concept of ‘compulsive sexual behaviour’ (CSB) is still controversially discussed in clinical practice as well as in the scientific literature. The term sexual addiction was introduced in the revised third version of the Diagnostic and Statistical Manual of Mental Disorders (DSM-III-R) (American Psychiatric Association 1987) and was then excluded from the DSM-IV (American Psychiatric Association 1994) due to a lack of empirical data. A diagnostic category for the phenomenon, named ‘Hypersexual Disorder’, was reintroduced in the developmental process of the DSM-5 (American Psychiatric Association 2013); however, it was excluded from the final version (Kafka 2014). In 2018, the World Health Organization’s (WHO’s) working group on Impulse Control Disorders proposed a new diagnosis of Compulsive Sexual Behaviour Disorder (CSBD) for consideration in the 11th revision of the International Classification of Diseases (ICD-11) (Kraus et al. 2018). CSBD is primarily characterised by ‘out-of-control’ sexual behaviours, which the affected person has repeatedly failed to control or to reduce and in which the affected person persists despite negative consequences in one or more important domains of functioning (e.g., socio-professional, personal). The affected person may suffer from psychological distress, or the sexual behaviours may involve risks of harming others. Distress entirely related to moral judgments and disapproval about sexual impulses, urges, or behaviours is not sufficient to diagnose CSBD.

Throughout the present manuscript, we will use the term CSB when sexual behaviours do not lead to personal distress or do not coincide with risks for harming others, or these factors were not assessed. We will refer to CSBD in cases in which sexual behaviours are accompanied by personal distress, social, professional or personal consequences or are associated with risks of harming others.

A phenomenon closely related to CSB is chemsex, and thus chemsex will also be addressed in these guidelines. CSB associated with paraphilias or paraphilic disorders will also be covered in the guidelines, in part given their frequent co-occurrence. After a review on the history, prevalence and clinical characteristics of CSB/CSBD and chemsex and the relationships between CSB/CSBD and paraphilias, we review the treatment options and propose guidelines for the pharmacological treatment of CSBD.

### History of CSBD

For centuries, humans have recognised and written about the idea that some people can develop dyscontrolled, excessive, or unregulated sexual behaviours (Briken 2020; Grubbs, Kraus, et al. 2020). Beginning at least with ancient Greece and the terms ‘nymphomania’ and ‘satyriasis’, there has long been recognition that people can become compulsive in sexual behaviour (Briken 2020). However, it was not until the 19th century that a more formal, medical understanding of such dysregulated sexual behaviours began to crystallise, with early writings on the topic from notable figures as Rush (1812) in the U.S. and later Krafft-Ebing (1965) in Europe. Periodic work on excessive appetitive behaviours, including excessive appetite for sex (Stekel 2013), continued to emerge over the early 20th century, and many early leaders in psychoanalysis opined about situations in which someone might develop an excessive sexual drive (Giugliano 2003). However, it was not until the mid-20th century that psychiatry and clinical psychology began to pay considerable and consistent attention to the notion that CSB should potentially be considered a distinct diagnosis.

In the late 1970s, Orford (1978) wrote about the implications of using theories of dependence, as they relate to addiction more broadly, to account for what he termed ‘excessive heterosexuality’. In this account, based on case reports and autobiographical accounts from individuals with such behavioural problems, Orford (1978) concluded that:

There is sufficient evidence to conclude that excessive heterosexuality exists as a social fact. There is enough testimony to the fact that some people have sought specialist help because they wished to restrain their sexual behaviour (heterosexual behaviour with adult partners) but were unable to do so. (p. 307)

Despite this seemingly sanguine take on the existence of CSB, Orford (1978) further noted, in seeking to understand CSB, there was little consensus regarding definitions, little knowledge regarding aetiology, general ignorance of social context, and a poor understanding of what constituted normal sexual behaviours. Ultimately, Orford concluded that, at the time of his writing, the evidence of CSB as an addictive disorder existed wholly in the form of anecdotes, rather than empirical or quantitative scientific evidence.

Despite the above-noted criticisms, clinical concerns about excessive sexual behaviour continued to appear in the published literature. In 1983, Patrick Carnes published the book, *Out of the Shadows: Understanding Sexual Addiction*, which introduced the concept of addiction to sexual behaviour to broader clinical audiences and popularised the idea that one could be addicted to sex (Carnes 1983). This initial work was based on clinical case reports and theoretical speculation, rather than empirical evidence, earning intense and largely valid criticism (Levine and Troiden 1988; Gold and Heffner 1998). Even so, interest in the notion of excessive, addictive, or compulsive sexual behaviour continued to grow throughout the late 1980s and early 1990s, in the form of case reports, theoretical speculations, and narrative accounts of this disorder (Pincu 1989; Coleman 1991; Goodman 1992). Additionally, as the HIV/AIDS crisis intensified over the 1980s and 1990s, interest in sexual compulsivity among gay and bisexual men also increased, resulting in quantitative studies of CSB in such populations (Kalichman et al. 1994; Kalichman and Rompa 1995; Benotsch et al. 1999).

Beginning in the late 1990s, secondary to advent of the internet and the widespread use of personal computers to access the internet, there was a boom of interest in sexual compulsivity, sexual addiction, CSB, pornography addiction, cybersex addiction, and a host of other similar constructs (Delmonico 1997; Cooper et al. 1998, 1999, 2000; Garcia and Thibaut 2010). The widespread use of the internet to access sexual materials such as pornography, sexual chatrooms, and forums for meeting offline sexual partners, spurred an exponential increase in research related to excessive and dyscontolled sexual behaviours (Grubbs, Kraus, et al. 2020).

This increase in empirical attention culminated in the proposal of Hypersexual Disorder (Kafka 2010) as a potential diagnosis in the DSM-5 (American Psychiatric Association 2013). This proposed diagnosis emphasised the potential for some people to exhibit dysregulated sexual behaviours, with a focus on patterns of symptoms quite similar to gambling and substance-use disorders (e.g., failed attempts to control or reduce behaviour, persistence in the behaviour despite negative consequences, persistence in the behaviour even when gaining little or no satisfaction from the behaviour). These criteria also specifically noted the excessive use of sexual behaviour to cope with stress or negative affective states as a symptom. Despite undergoing a successful field trial (Reid, Carpenter, et al. 2012), the diagnosis was ultimately excluded from the DSM-5 (Kafka 2014). Even so, a very notable increase in empirical literature related to excessive, addictive, or out-of-control sexual behaviours was generated by this consideration (Grubbs, Kraus, et al. 2020), ultimately culminating in a novel diagnosis proposed and included in the ICD-11, as we discuss below.

### Definition of CSBD

In the years following the exclusion of Hypersexual Disorder from the DSM-5, research related to excessive and dyscontrolled sexual behaviour continued to increase. Based on the preponderance of new evidence, in 2018, the WHO’s working group on Impulse Control Disorders proposed a new diagnosis of CSBD for consideration in the ICD-11 (Kraus et al. 2018). After extensive consideration and public commentary (Fuss, Lemay, et al. 2019), this disorder was ultimately included in the ICD-11 as an impulse-control disorder.

Similar to past conceptualizations of sexual addiction (Carnes 1983), sexual compulsivity (Coleman 1991; Bancroft and Vukadinovic 2004; Bancroft 2008), and hypersexual disorder (Kafka 2010), CSBD is primarily concerned with poorly controlled sexual behaviours. The affected individual has made repeated unsuccessful attempts to control or reduce the behaviours and persists in the behaviour despite negative consequences across multiple life domains. The full diagnostic criteria for this disorder are available in Table 1.

**Table 1.** Diagnostic criteria for Compulsive Sexual Behaviour Disorder for ICD-11.

Essential (required) features for CSBD • 1. A persistent pattern of failure to control intense, repetitive sexual impulses or urges resulting in repetitive sexual behaviour, must be manifested in one or more of the following: ^ 1a. Engaging in repetitive sexual activities has become a central focus of the person’s life to the point of neglecting health and personal care or other interests, activities, and responsibilities (yes/no). ^ 1b. The person has made numerous unsuccessful efforts to control or significantly reduce repetitive sexual behaviour (yes/no) ^ 1c. The person continues to engage in repetitive sexual behaviour despite adverse consequences (e.g., repeated relationship disruption, occupational consequences, negative impact on health) (yes/no). ^ 1d. The person continues to engage in repetitive sexual behaviour even when the individual derives little or no satisfaction from it (yes/no). • 2. The pattern of failure to control intense, sexual impulses or urges and resulting repetitive sexual behaviour is manifested over an extended period (e.g., 6 months or more) (Must be met) • 3. The pattern of repetitive sexual behaviour causes marked distress or significant impairment in personal, family, social, educational, occupational, or other important areas of functioning (Must be met). *Note for rule out*. Distress that is entirely related to moral judgments and disapproval about sexual impulses, urges, or behaviours is not enough to meet this requirement.

Notably, CSBD differs from past conceptions of dysregulated sexual behaviour (i.e., Hypersexual Disorder) on a few key points. Primarily, CSBD no longer lists the use of sexual behaviour to cope with negative affect or stress as a key feature or criterion for the disorder. This omission has been controversial, with some commentaries noting that the disorder could consider including such behaviour as a criterion, although similar differences between the DSM-5 and ICD-11 have been noted for other conditions like gambling disorder (Gola et al. 2020). More generally, which behaviours may be considered as core symptoms of a disorder versus underlying psychological processes has been debated for psychiatric disorders more generally (Brand, Rumpf, King, et al. 2020). Additionally, CSBD is the only diagnosis to specifically note that distress related to moral judgments and disapproval of sexual impulses, urges, or behaviours alone are not enough to warrant receiving the diagnosis. This specific note is unique to CSBD and may reflect relationships between sexual behaviour and personal values and morality, which we will return to later in our considerations of the psychosocial correlates of CSBD. However, it should be noted that the concept of moral incongruence may not be specific to CSBD and may apply, for example, to a range of addictive disorders and behaviours (Brand et al. 2019; Lewczuk et al. 2021).

## Prevalence of CSB in the general population

One problem in determining the precise prevalence of CSB comes with the considerable variance in study findings. Previous studies have varied concerning the specific definition of CSB and concerning the characteristics of the studied samples. In one of the first studies, Alfred Kinsey assessed the number of orgasms per week in a large sample of men in the 1930s and 1940s. He found that 7.6% of men younger than 30 years of age reported more than seven orgasms a week for at least five consecutive years, a value that was used as an indicator for CSB in later studies as well (Kinsey et al. 1948; Klein et al. 2015). Klein and colleagues (2015) determined in a sample of more than 8000 men from Germany that 12.1% showed CSB based on such a cut-off of more than seven orgasms per week. Laumann et al. (1994) reported that 7.6% of males (*n* = 1320) engaged in partnered sex ≥4 times/week for at least 1 year, which they viewed as an indicator for CSB.

Långström and Hanson (2006) also found a prevalence of CSB of 12.1% in a representative sample of Swedish men. They used a self-constructed measure including eight different behaviours and defined CSB statistically (e.g., times of masturbation during last month, number of sexual partners, ever having had group sex, etc.). In that sample, 6.8% of the included women showed CSB. In another Swedish online sample of 1913 participants (65.3% women), 5% of women and 13% of men reported at least some problems with viewing sexual content on the internet measured with a self-constructed five item scale, while 2% of women and 5% of men reported they had serious problems (Ross et al. 2012). In an online survey of U.S. adults (*N* = 2075), 11% of men and 3% of women agreed to the statement that they were addicted to pornography (Grubbs et al. 2019).

In summary, most studies have found prevalence rates between 8% and 13% for men and between 5% and 7% for women; however, distress or interpersonal problems due to CSB were not assessed strictly in these previous studies. Meanwhile, many clinicians and researchers estimate the prevalence of CSB qualifying for a diagnosis of a psychiatric disorder (e.g., CSBD) to be between 3% and 6% in the general population (Odlaug et al. 2013; Kraus et al. 2018).

A recent representative study from the U.S. found that 10.3% of the men and 7% of women from their sample reported clinically relevant levels of distress or interpersonal impairment related to sex (Dickenson et al. 2018). Using latent profile analysis on the newly developed CSBD-19 questionnaire in a sample of over 9300 men from three countries, 2.8% of the included participants could be allocated to the high-risk group which corresponded most likely to a clinically relevant level of CSB (Bőthe et al. 2020). Castro-Calvo et al. developed a composite index based on three well-known validated scales (the Hypersexual Behaviour Inventory-19 (HBI-19), the Sexual Compulsivity Scale (SCS), and the Sexual Addiction Screening Test (SAST)), thereby covering a range of CSBD symptoms and severity. Using a cluster-analytic approach on two samples comprising in total 2899 participants (sample 1: 1581 university students, 56.9% female; sample 2: 1318 community members, 43.6% female), 9.1% could be classified as meeting criteria for CSBD, of whom 70% were men (Castro-Calvo et al. 2020). In the so far largest study assessing the prevalence of CSBD defined and operationalised according to the ICD-11 guidelines including 4633 individuals from the general population in Germany (50.5% male), a lifetime CSBD prevalence of 4.9% [95% CI = 3.9–6.1] in men and of 3.0% [95% CI = 2.3–3.9] in women was found. Furthermore, a 12 month-prevalence of 3.2% in men and of 1.8% in women was reported (Briken et al. 2022).

It has been hypothesised that in some populations CSB would be found at a higher rate compared to the general population; for example, in men convicted of a sexual offence or those engaging in chemical sex or chemsex (see sections ‘CSBD and paraphilic disorders’ and ‘Prevalence and characteristics of chemsex use,’ respectively).

Interestingly, some differences in the prevalence or type of CSB have been reported when gender or sexual orientation was considered. In a study conducted in men (*n* = 64) and women (*n* = 16) with self-identified CSB, the most prevalent problematic sexual behaviour reported by men was the use of pornography in 82% of cases as compared to 50% of women, whereas the most frequently reported sexual behaviour in women was engagement in sex with consenting adults (88%), as compared to 36% in men (Öberg et al. 2017). A population-based study (*n* = 18,034) suggested that non-heterosexual men and to a lesser extent non-heterosexual women (i.e., gay, bisexual) and transsexual and queer individuals had the highest prevalence of CSB (frequency of masturbation, number of sexual partners or frequency of pornography viewing), including the highest scores on the HBI-19 (Bőthe, Bartók, et al. 2018). However, interestingly and contrary to other findings (e.g., Blum et al. 2020), Gleason et al. (2021) estimated a prevalence of 7.9% with clinically significant CSB among gay men in the U.S. – not higher than the prevalence in the general population.

Furthermore, own experiences of sexual abuse during childhood have been positively related to CSB; however, this association was stronger in men than in women. The few clinical studies addressing adverse childhood events in CSBD patients have suggested overall estimates of 30% of CSBD patients having experienced childhood sexual abuse (Slavin et al. 2020).

## Co-occurring conditions

### Co-occurring psychiatric disorders

CSBD may co-occur with other psychiatric disorders; however, CSB may also occur as a symptom of another psychiatric disorder, complicating differential diagnosis in some patients. In cases in which one believes CSB to be a symptom of another disorder, no diagnosis of CSBD should be made. Only in cases in which CSB appear to exist non-secondary to other disorders should one consider diagnosing a CSBD. For example, CSB may occur during the course of a substance-use, affective, psychotic or neurological disorder. Additionally, some medications (e.g., dopamine agonists) carry warnings regarding the possible emergence of CSB. In such cases, careful clinical diagnosis is warranted.

Co-occurring psychiatric disorders are common in individuals with CSB (Kaplan and Krueger 2010; Ballester-Arnal et al. 2020). Kafka and Prentky (1994) assessed a sample of 26 men seeking treatment for a principal diagnosis of paraphilia-related CSBD and found that 80.8% qualified for a co-occurring mood disorder and 46.2% for a co-occurring anxiety disorder. Comparably, pronounced co-occurrence rates were also reported by Kafka and Hennen (2002) in another sample of 32 men with CSBD. In that latter study, all included participants could be diagnosed with at least one additional axis I disorder, and 68.7% were diagnosed with dysthymic disorder, 40.6% with major depression, 25% with social phobia, and 15.6% with generalised anxiety disorder (Kafka and Hennen 2002).

More recent and larger studies from different cultural backgrounds also support these results (Weiss 2004; Schultz et al. 2014; Scanavino et al. 2018; Engel, Veit, et al. 2019; Briken et al. 2022). In a study assessing 72 outpatients (94.4% male) seeking treatment for CSB in France, 63.9% were diagnosed with major depressive disorder, 33.3% with generalised anxiety disorder, and 41.7% with social phobia (Wéry et al. 2016). It has been suggested that symptoms of emotional dysregulation which can frequently be found in mood and anxiety disorders but also in men suffering from CSB may be a uniting element between these different diagnostic entities (Lew-Starowicz et al. 2020). Many patients describe that CSBs help to relieve negative affect, to cope with affective symptoms of another psychiatric disorder, or to face adverse life events (Ross et al. 2012; Wéry et al. 2016). However, CSBs are also frequently followed by negative mood states, relationship problems, financial difficulties or job loss, factors that can in turn negatively influence symptoms of co-occuring affective or anxiety disorders (Dhuffar et al. 2015; Koós et al. 2021). This may constitute a vicious cycle that contributes to the maintenance of CSB and co-occurring symptomatology.

Regarding bipolar disorders, associations are complex. In clinical practice, CSB is a frequent symptom associated with mania and hypomania (Jamison et al. 1980; Carta et al. 2014; Kopeykina et al. 2016). A meta-analytic review on the clinical characteristics of children and adolescents with a bipolar disorder found that CSB appear in about 31–45% of adolescents during a manic episode (Kowatch et al. 2005). In adults, comparable studies are lacking. However, a recent meta-analysis found that in 17% of patients with bipolar disorder (95% CI 6%–33%), CSB has occurred as a prodromal symptom prior to the first manic episode, and CSB is among the most frequent symptoms preceding recurrent bipolar mood episodes (van Meter et al. 2016). In bipolar patients, however, the question arises whether CSB should really be seen as a comorbid disorder or whether CSB in these cases is more a symptom of the hypomanic/manic episodes (associated with more general behavioural disinhibition) and subsides as soon as the manic episode has remitted.

Previous research has also related compulsivity to CSB (Carnes 1983, 1991; Stein 2008; Bőthe, Koós, et al. 2019). Compulsivity is reflected in ‘the performance of repetitive and functionally impairing overt or covert behaviour without adaptive function, performed in a habitual or stereotyped fashion, either according to rigid rules or as a means of avoiding perceived negative consequences’ (Fineberg et al. 2014). Compulsivity can present itself, for example, in repetitive behaviours helping to temporarily reduce distress or eliminate fear, suggesting a possible overlap with CSB. Compulsivity may also reflect behaviour that is done habitually when there is little or no pleasure associated with the behaviour, as reflected in the criteria for CSBD (Kraus et al. 2018). Bőthe and colleagues found a small but significant association between compulsivity and CSB in a sample of 13,778 men and women from the general population (Bőthe, Koós, et al. 2019). In the same way, Black et al. (1997) had previously found 42% of individuals with CSB reported the presence of intrusive and repetitive sexual fantasies, and 67% presented repetitive sexual behaviours, which were initially resisted and were followed by negative self-esteem. On the other hand, in a recent study with 539 outpatients with a current obsessive-compulsive disorder (OCD) (51.8% female), only 5.6% were also diagnosed with lifetime CSBD and 3.3% with current CSBD (Fuss, Briken, et al. 2019), suggesting that the prevalence of CSB in OCD patients is comparable to the prevalence of CSB in the general population. In patients with CSB, rates of OCD ranged between 2% to 14% (Scanavino et al. 2013; Kafka 2015).

Studies addressing the co-occurrence of eating disorders and CSB are scarce up to now. However, as impulsivity and deficits in emotion-regulation can frequently be found in patients with eating disorders as well as in individuals with CSB, there could be a possible relationship between these clinical conditions (Waxman 2009; Brockmeyer et al. 2014; Bőthe, Koós, et al. 2019). Despite this theoretical overlap though, in the study by Wéry et al. (2016), only one of the participants with CSB was diagnosed with anorexia nervosa and none with bulimia nervosa. In women with an eating disorder, those with binge-purging types showed rates of CSB comparable to the control group, while in women with a restricting type, lower rates of CSB were found. Further analyses showed that this relationship was mainly mediated by symptoms of emotion regulation and experiences of sexual abuse during childhood (Castellini et al. 2020). Studies assessing the relationship between CSB and eating disorders in men are to our knowledge missing so far.

CSB also frequently co-occurs with substance-use disorders (SUDs). In the study of Kafka and Hennen (2002), more than 40% of patients with CSB were diagnosed with an additional SUD, while alcohol use disorder was found most frequently (see Reid and Meyer (2016) for a review on co-occurring SUDs in CSB patients). However, only the abuse of cocaine was observed more commonly in men with CSB compared to men with paraphilias (Kafka and Hennen 2002). In individuals with SUDs, only those with cocaine use disorders reported CSB significantly more frequently, with no differences in CSB found for patients with alcohol, cannabis, amphetamine, opiate or benzodiazepine use disorders (Stavro et al. 2013). Cocaine and amphetamine increase dopamine and noradrenaline in the brain, and it has been suggested that especially dopamine may have general disinhibitory effects and specific disinhibitory effects on sexual behaviour (Dominguez and Hull 2005; Pfaus 2009).

CSB also co-occurs with behavioural addictions (note that some researchers and clinicians view CSBD, especially excessive online pornography consumption, as a behavioural addiction, e.g., Brand, Rumpf, Demetrovics, et al. 2020; Gola et al. 2020; Karila et al. 2014). In a study from Brazil including 458 people with gambling disorder, 6.4% reported CSB that fulfilled criteria to be considered as a psychiatric disorder (Tang et al. 2020). In the other direction, a prevalence ranging between 2% and 8% of behavioural addictions (e.g., gambling disorder, gaming disorder, or compulsive buying-shopping) has been reported in samples presenting with CSB (Raymond et al. 2003; Scanavino et al. 2013; Wéry et al. 2016).

Single studies suggest that individuals with CSB may demonstrate elevated attention-deficit/hyperactivity disorder (ADHD) symptomatology; however, the symptomatology may not reach diagnostic levels (e.g., Engel, Veit, et al. 2019; Bőthe, Tóth-Király, et al. 2019). In earlier studies, rates of ADHD between 20% and 27% have been reported in men with CSB (Kafka and Hennen 2002; Reid 2007; Reid, Carpenter, et al. 2011), whereby the inattentive subtype was observed more frequently than the impulsive or combined subtypes. Depending on the diagnostic threshold for ADHD, a co-occurrence rate for CSB between 5% and 12% was reported in one previous study (Bijlenga et al. 2018). In an online study from Germany with 139 adults with ADHD (*n* = 89 women), about a quarter (24.5%) reported CSB, whereby 14.6% of women and 45.5% of men were affected. Despite these high co-occurence rates, CSB was not found significantly more often than in adults without ADHD in that study (Gregorio Hertz, Turner, et al. 2022). It was suggested that increased impulsivity and problems with emotion regulation might be underlying mechanisms explaining the overlap between both clinical conditions (Soldati et al. 2021; Gregorio Hertz, Turner, et al. 2022). However, so far only few studies exist looking precisely at the relationship between ADHD and hypersexuality, and ADHD should be systematically researched and assessed clinically in adults with CSBD.

Similar considerations hold for CSB and autism spectrum disorders. To date, few studies have assessed CSB in individuals with autism spectrum disorders, and most are case reports involving individuals with mild to severe cognitive impairments (Nguyen and Murphy 2001; Müller 2011; Hergüner et al. 2012; Shahani 2012; Deepmala and Agrawal 2014). As an increased rate of CSB, especially excessive masturbation, has repeatedly been reported in individuals with cognitive impairments, it is unclear whether CSB may be due to autism-specific symptoms or a general disinhibition following cognitive impairment (Wallace and Safer 2009). In a sample of 90 individuals diagnosed with high-functioning autism, 23.3% showed signs of CSB compared to 6% in the control group from the general population (Schöttle et al. 2017). It was suggested that repetitive behaviours, special interests, and peculiarities in sensory perception might lead to a higher rate of CSB; however, because of the frequently observed deficits in social communication, CSB may mainly manifest in masturbation in individuals with autism spectrum disorders (Turner et al. 2017).

Concerning CSB and psychotic disorders, few case reports exist regarding relationships between conditions (e.g., Volpe and Tavares 2000; Kar and Dixit 2019). Furthermore, most case reports rather relate treatment with certain antipsychotics (especially aripiprazole, but also olanzapine, clozapine, and risperidone) with CSB in patients with psychotic disorders; however, systematic studies on this topic are missing to our knowledge (Cheon et al. 2013; Reddy et al. 2018; Thomson et al. 2018; Stefanou et al. 2020). It has been suggested that partial agonistic effects at dopamine D2-like receptors may account for CSB in patients treated with aripiprazole (Stefanou et al. 2020). However, such a relationship is based on case reports and has not been substantiated in large-scale epidemiologically sound studies. Although there are reports that CSB may improve or cease when antipsychotic treatment is withdrawn or replaced with another antipsychotic, more studies appear warranted.

Besides high rates of Axis I disorders, previous research has also found considerable prevalence rates of personality disorders in patients with CSB (Carpenter et al. 2013; Ballester-Arnal et al. 2020). Cluster B personality disorders, as well as an obsessive-compulsive personality disorder, seem to co-occur especially often with CSB (Ballester-Arnal et al. 2020). It was suggested that patients diagnosed with borderline personality disorder can also show signs of CSB, especially risky sexual behaviours (Sansone et al. 2008; Sansone and Sansone 2011). However, most previous studies have operationalised CSB only by the number of sexual partners and have neglected other forms of sexual behaviour. In a sample of 132 men diagnosed with a CSBD according to the proposed DSM-5 criteria for hypersexual disorder, 48% met the diagnostic criteria for borderline personality disorder using the Structured Clinical Interview for DSM-II (SCID-II) screening questionnaire. However, none qualified for an actual diagnosis following the administration of the SCID-II interview, questioning the overlap between CSB and borderline personality disorder (Carpenter et al. 2013). The personality disorder most prevalent in that sample of men showing CSB was a narcissistic personality disorder with a prevalence of 8%, thereby exceeding the suggested prevalence for the general population, which is suggested to be between 1% and 2% (Volkert et al. 2018). Individuals high in narcissism and especially sexual narcissism usually report a high number of sexual partners, a high rate of infidelity (Jonason et al. 2009; Klein et al. 2020), and a low overall relationship commitment (Campbell and Foster 2002), underscoring the possible overlap with CSB. Whether or not an antisocial personality disorder is related to CSB is unclear so far. In one study among college students, at least the personality construct of psychopathy was related to all facets of CSB (Kastner and Sellbom 2012). In a large sample of more than 8000 men living in Germany, a small but statistically significant correlation between CSB and antisocial behaviours was found, and both factors were related to the consumption of child-sexual-abuse imagery (child pornography), suggesting that the combination of CSB and antisociality could be a risk factor for sexual offending (Klein et al. 2015). However, more research is needed on this topic.

In summary, CSB frequently co-occurs with mood, anxiety, substance-use, addictive, impulse-control and particular personality disorders. However, more research considering CSBD and not only CSB is needed. In other psychiatric disorders, for example, bipolar disorder, ADHD, autism spectrum disorders or some other personality disorders, CSB may rather occur as a symptom and not so frequently as a comorbid disorder.

### CSB and neurological diseases

Frontal brain injuries point towards disinhibition and temporal lobe lesions may promote sexual drive (Kühn and Gallinat 2016). Neurological diseases may be confused with disorders of sexual behaviour or can be associated with them (Krueger and Kaplan 2000). Delirium, temporal or frontal lobe lesions, post-brain injury states (Britton 1998; Gaudet et al. 2001; Sander et al. 2013; Turner et al. 2015), seizure disorders, multiple sclerosis, dementia – particularly fronto temporal dementia (Mendez and Shapira 2013), Wilson’s disease (Volpe and Tavares 2000), Kleine-Levin syndrome (Bonnet et al. 1996) and Klüver-Bucy syndrome (Ott 1995) may present with CSB as a prominent symptom. Intellectual disability may also be associated with CSB (Davies 1974; Mann and Travers 2020). Similarly, CSB may be observed in 4–30% of men with Huntington’s disease and in 2–25% of women in association with irritability, obsessive-compulsive or perseverative behaviours (Fedoroff et al. 1994; Craufurd et al. 2001; Jhanjee et al. 2011).

Patients with Parkinson’s disease can also develop different challenging behaviours in the later course of the disorder, including pathological gambling, binge-eating, compulsive buying, and CSB (Evans et al. 2009). The aetiologies of these behaviours are complex. In patients with Parkinson’s disease receiving dopaminergic agonists, such as pramipexole, ropinirole, pergolide, rotigotine, apomorphine or bromocriptine, some may experience CSB (Seeman 2015). In one of the largest studies conducted to date (the DOMINION study that involved over 3000 patients with Parkinson’s disease and actively screened for problems with gambling, eating, shopping/buying and sexual behaviours), one or more impulse-control behaviour was observed in 13.6% of patients, with those receiving dopamine agonists, receiving levo-dopa, living in the United States (versus Canada), being of younger age, being unmarried, currently smoking cigarettes, and having a family history of gambling problems being more likely to have one or more impulse-control problems (Weintraub, Koester, et al. 2010). Subsequent analyses of the DOMINION data suggested a link between amantadine and impulse-control problems and overlapping and shared characteristics linked to individual impulse-control problems (Weintraub, Sohr, et al. 2010; Voon, Sohr, et al. 2011). In a separate open study of people with Parkinson’s disease, at a mean follow-up period of 29 months, 83% of patients with impulse-control problems were reported to no longer meet criteria after cessation or reduction of the dopamine agonist, without a worsening in motor symptoms reported (Witjas et al. 2012). Temporary replacement of pramipexole or ropinirole with bromocriptine may provide relief or reversal of the impulsive behaviour associated with either pramipexole or ropinirole (Seeman 2015). However, the open-label nature of these studies, concerns regarding reporting and expectations, and the lack of specificity to CSB rather than impulse-control problems more generally suggest the need for additional study (Potenza 2013, 2018; Gendreau and Potenza 2014, 2016).

CSB has been reported in people with other disorders treated with dopamine agonists or levodopa, such as restless legs syndrome, multiple system atrophy, progressive supranuclear palsy, pituitary adenoma or fibromyalgia, particularly with higher dopamine agonist doses (Martinkova et al. 2011). Of 140 patients with restless legs syndrome, eight were receiving dopamine agonists and among them, two reported CSB (Voon, Schoerling, et al. 2011). A 53-year-old man with macroprolactinoma was reported to have experienced severe CSB after cabergoline (a dopamine agonist) was started (Martinkova et al. 2011). Finally, hyperandrogenic states or pituitary dysfunction may also be associated with CSB.

There are several case reports of various medications that have been tried to reduce CSB in the setting of dopamine-related pharmacotherapies. The atypical antipsychotics, serotoninergic antidepressants, histaminergic (H2 receptors) antagonists or antiandrogens (cyproterone acetate, medroxyprogesterone acetate or GnRH agonists may rarely be used in cases of aggressive sexual behaviour) have been reported to be of some benefit. Antiepileptics, such as topiramate, have been reported to be linked to the resolution of impulse control disorders such as CSB in several cases. The efficacy of cholinesterase inhibitors in CSB associated with dementia is controversial. Family involvement and psychotherapy may also be beneficial (Zhang et al. 2021). Overall, data are limited and largely linked to case reports.

### CSBD and paraphilic disorders

Given similarities between paraphilic disorders and CSB, some authors have speculated that both disorders could be considered as obsessive-compulsive-spectrum disorders or compulsive-impulsive disorders (Rösler and Witztum 2000; Beech and Mitchell 2005). Multiple neurological disorders involving frontal or temporal regions have been associated either with paraphilic behaviours and/or CSB (Thibaut et al. 2020). Finally, Kafka (2001) proposed that CSB should be considered as a paraphilia-related disorder (PRD). He recommended making a distinction between culturally normative sexual behaviours (e.g., masturbation, pornography use) and paraphilic sexual behaviours (i.e., sexual behaviour involving nonhuman objects, the suffering or humiliation of oneself or one’s partner or children/nonconsenting partners) to identify either a PRD, paraphilic disorder or both. According to Briken et al. (2007), a comorbidity is present only if PRD and/or paraphilias are independent and not related to symptomatic progression.

The comorbidity of both disorders remains poorly understood, and links with sex offending are even more complex. For example, according to Finkelhor’s model (Finkelhor 1984), four stages are reported before a sexual offence. The individual must go through all stages before a sex offence: (1) the individual must be motivated (paraphilic fantasies: e.g., emotional or sexual attraction to a child or to a non-consenting adult); (2) the individual must overcome internal inhibitions (e.g., social norms); (3) the individual must overcome external inhibitions (e.g., remove the child from others who protect him or her); (4) the individual must overcome the victim’s resistance (hold; grip and/or physical violence). Paraphilic fantasies, CSB, past history of sexual abuse, early exposure to violent pornography or frequent exposure to child-sexual-abuse imagery (child pornography) may increase the frequency of occurrence of this vicious cycle which becomes compulsive and increases the risk of offending. A better knowledge of these four steps may help patients to identify risk triggers and manage them in order to prevent offences.

#### Prevalence of paraphilias or paraphilic disorders in patients showing CSB

Some studies have been conducted in the general population. As stated above, Långström and Hanson (2006) have studied 18–60 year-old participants from a representative, non-clinical Swedish population and found that high rates of impersonal sexual activity were associated with paraphilic interests such as voyeurism, exhibitionism, sadism, and masochism (odds ratios of 4.6–25.6 were reported in both sexes). Dawson et al. (2016) asked 305 men and 710 women to complete an online survey. Men reported significantly less repulsion and/or more arousal to many paraphilic activites compared to women, and sex drive mediated most of the sex differences in paraphilic interests. In another online survey involving 1194 participants (including 564 women), 13.1% of women and 45.4% of men were considered as having CSB. Severity of CSB was associated with elevated rates of sexual fantasies involving coercion and a high rate of actual sexual coercion in both genders (Engel, Veit, et al. 2019). Klein et al. (2015) reported an association between self-reported child sexual abuse imagery consumption and sex drive, sexual fantasies involving children, and antisociality in a large non-clinical male German sample (8718 males; online study). Whether child-sexual-abuse imagery consumption is linked to previous paedophilic fantasies or to previous CSB requires additional studies. Several authors have identified a relationship between frequent pornography use and child-sexual-abuse imagery consumption (Svedin et al. 2011; Seigfried-Spellar and Rogers 2013; Seto et al. 2015). Moreover, child-sexual-abuse imagery pop-up windows may appear when people often use adult pornography, and people may be attracted to prohibited novelties. Early and easy access to pornography may also increase the subsequent risk of compulsive use of pornography (Hall 2013).

In rare cases, CSB may be associated with indecent exposure or rape without previously known paraphilic disorders. The incidence of sexual offending or paraphilic disorders in patients showing CSB is poorly known. Concurrent paedophilia or sexual urges involving pre-pubertal children was reported by 9% of a male sample (*n* = 78), in line with previous reports of 6% in a clinical cohort of CSBD patients (*n* = 36) (Black et al. 1997). In a small sample of 72 self-identified patients with CSBD seeking treatment in a dedicated outpatient unit, 60.6% of patients reported at least one paraphilia: mainly voyeurism, exhibitionism; paedophilia was reported in 14% of cases (Wéry et al. 2016). Engel, Veit, et al. (2019) have compared 50 men with CSBD against 40 individuals without CSBD. Paraphilias like exhibitionism, voyeurism, masochism, sadism, fetishism, frotteurism or transvestism were more prevalent in men with CSBD as compared with men without CSBD (47% vs. 3% respectively). Men with CSBD were also more likely to report sexually coercive behaviour (70% vs. 21%) and having consumed child-sexual-abuse imagery at least once in their lives (81% vs. 0%). Novelty-seeking has also been found to be associated with CSB (Banca et al. 2016), and fantasies of sexual coercion or child sexual interest may function as new, sexually interesting, stimuli in people with CSB.

In summary CSB/CSBD may be a risk factor for acting out sexually coercive behaviour and viewing child-sexual-abuse imagery. However, further studies are needed in this important field.

#### Prevalence of CSBD and pornography use in individuals with paraphilias or paraphilic disorders

In Kafka and Hennen’s (2003) study, paraphilias were associated with CSB in 72–80% of 120 evaluated men seeking treatment for paraphilias or paraphilia-related disorders. In a sample of 60 men convicted for the possession of child-sexual-abuse imagery, 33% were diagnosed with CSBD after extensive psychiatric assessment (Krueger et al. 2009). Comparably, Marshall and Marshall (2006) found a prevalence of CSBD in 35% of 40 sexually-offending individuals from Canada. Somewhat smaller numbers were reported by Kingston and Bradford (2013) in a sample of 586 men convicted of a sexual offence. In their sample, only 12% fulfilled the criteria for CSB defined as more than seven orgasms per week. In a recent study, a prevalence of 6.6% for a CSBD based on the diagnostic criteria for hypersexual disorder proposed by Kafka (2010) was found (Gregorio Hertz, Rettenberger, et al. 2022). Thereby, CSBD was predictive for contact sexual offending and even added incremental predictive validity beyond the joint Static-99 and Stable-2007 (Gregorio Hertz, Rettenberger, et al. 2022). Finally, in a population of 341 individuals who molested children, Kingston et al. (2008) reported that the frequency of pornography use was a risk factor for higher-risk individuals whereas the content of pornography (e.g., paedophilic content) was a risk factor for all individuals.

Thus, in summary, recent studies suggest that the prevalence of CSB or CSBD in people with paraphilias or paraphilic disorders could be comparable to the prevalence reported in the general population. However, CSB may be considered as a risk factor for sexual reoffending in men who have been convicted for a sexual offence (Mann et al. 2010; Kingston and Bradford 2013).

Some studies have also been conducted in the general population. Turner et al. (2016) conducted an anonymous online survey on 8649 German men; 0.4% were men who abused children while working with children (CSAW); 1% abused children while not working with children and 9.4% worked with children and had not abused any children (non-CSAW). CSAW spent more time thinking about sexuality and using pornography every day. They also had a higher aggregated sex drive index compared to non-CSAW. In a population of 775 Italian university students (243 men, 532 women), Castellini et al. (2018) reported that half of men and 41.5% of women reported at least one paraphilic behaviour. Men reported a higher prevalence of voyeurism, exhibitionism, sadism, and frotteurism while women reported a higher prevalence of fetishism and masochism (Castellini et al. 2018). All paraphilic groups except for the transvestism group reported more CSB, as compared with individuals without any paraphilic behaviour. Finally, Hald et al. (2010) conducted a meta-analysis and concluded that a positive association was found between attitudes supporting violence against women in real-life settings and pornography use, particularly sexually violent pornography. In summary, paraphilic fantasies are frequently associated with CSB in the general population.

#### Common risk factors between CSB and sex offending

Attachment and trauma issues are frequently observed in both individuals showing CSB and in patients with paraphilic disorders. Childhood sexual victimisation is more prevalent in people with paraphilic disorders (especially paedophilic disorders: range of 28–93%) as compared to the general population (15%) (Jespersen et al. 2009). A past history of sexual abuse may increase the potential for later child sexual abuse in males. However, the claim that sexual abuse ‘causes’ paedophilia has been disputed (Leach et al. 2016); only a proportion of sexually abused children develop a paedophilic disorder (Nunes et al. 2013). This association might be moderated by genetic or environmental factors (e.g., experiences of neglect in childhood, lack of parental supervision, intrafamilial violence and poor parent-child attachment) (Marshall et al. 2000; Salter et al. 2003) as well as specific characteristics of the experienced abuse (duration, timing, use of violence, penetration, relationship to the perpetrator and having perpetrators of both sexes) (Burton et al. 2002). The exact mechanism by which history of being sexually abused increases the likelihood of developing a paedophilic disorder remains unknown: learned associations; imitation; anger; frustration; revenge; or a desire to be punished are mechanisms that have been proposed. Becker (1998) suggested a probable basis for the development of a ‘deviant’ sexual arousal pattern in children with a past history of sexual abuse. They assume that ‘deviant’ sexual arousal and behaviour are learned in individuals through modelling and conditioning experiences. This is consistent with animal studies showing that the impact of damage to the hypothalamus on sexual functioning depends on the existence of prior sexual experience (for review, Chagraoui and Thibaut 2016).

Onset of sexual abuse prior to 7 years of age was also significantly associated with CSB in a sample of 449 mentally-ill youths (McClellan et al. 1996). Psychological abuse in childhood and adolescence, especially by a father, was found to be the most prominent predictor of subsequent compulsive-sexual thoughts and behaviours (Kingston et al. 2017).

Finally, early exposure to sex, pornography and/or sexual violence may also contribute to sex offending or CSB (Jespersen et al. 2009; Hall 2013). Given the increasing prevalence of children’s exposure to pornography in Western countries, studies are urgently needed.

## CSB and chemical sex

### Definition and history of the term chemical sex

Cocaine, ecstasy (methylene-dioxy-metamphetamine or MDMA), alkyl nitrites (‘poppers’), ketamine, synthetic amphetamines (also called ‘speed’) or alcohol have been used by the gay club scenes for many years (Bourne et al. 2015). The term ‘chem’ came from ‘chemicals’ and was introduced for the voluntary use of methamphetamine and gamma hydroxybutyrate or gamma butyrolactone (GHB/GBL) by gay men in their communications with their dealers. The use of mephedrone (a synthetic cathinone) started in London in 2006. ‘Chem’ use in association with sexual environments and networks was defined as ‘chemsex’ and has been used to define some gay communities since 1999 (Stuart 2019). This societal phenomenon was exacerbated by the increasing use of dating apps.

According to the compounds, these illicit drugs can be snorted, swallowed, smoked, or injected (with the last colloquially referred to as ‘slamming or slam sex’) or used via anal plug. According to Stuart (2019), the word ‘chemsex’ was helpful to identify this new public health concern, to consider chemsex apart from other forms of drug use or abuse, and to respond more effectively. Even if chemsex has been mainly studied in men who have sex with men (MSM) populations, chemsex behaviours are also being observed in the general population (Malandain et al. 2020). Chemsex is defined by the voluntary use of specific drugs before or during planned sex to initiate, facilitate, prolong, and/or intensify sexual activity and pleasure. The effects sought in the practice of chemsex are mainly as follows (Ahmed et al. 2016; Deimel et al. 2016; Weatherburn et al. 2017; Bui et al. 2018; Glynn et al. 2018; Hammoud et al. 2018; Lim et al. 2018; Tan et al. 2018; Hibbert et al. 2019):

- increased desire, sexual arousal and pleasure;
- increased duration of sexual activities, with some individuals describing sexual activities lasting several days;
- search for a disinhibitor and facilitator effect, allowing sexual intercourse more easily;
- search for acts that would not be performed without the use of substances, such as practices described as ‘hard;’
- experiencing the social nature of taking psychoactive substances, in order to fight against a feeling of loneliness, but also to increase self-confidence, attractiveness, emotional intimacy or feeling more in control of one’s sex life;
- to fight against a weakening of desire.

### Illicit drugs associated with chemsex

The most commonly used illicit drugs for chemsex in Western Europe are crystal methamphetamine (also called ‘ice or crystal meth’), gamma-hydroxybutyrate or, its prodrug gamma-butyrolactone (GHB/GBL; also called ‘blue nitrate’ or ‘G’ preparation; a GABA_B_ receptor agonist), mephedrone (4-methylmethcathinone [4MMC]), and, less often, other synthetic cathinones, cocaine, ketamine (2-(2-chlorophenyl)-2-(methylamino)cyclohexan-1-one; a NMDA receptor antagonist), or alkyl nitrites (also called poppers which are potent vasodilators). Cathinone is a naturally occurring beta-ketone amphetamine analogue (also called bk-amphetamine). Synthetic cathinones (such as mephedrone) are derivatives of this compound. Besides their amphetamine-like properties, they can modulate serotonin and have distinct psychoactive effects (Prosser and Nelson 2012).

Among 397 MSM having chemsex in the United Kingdom (UK), 74.1% reported using two or more drugs (mostly GHB/GBL in 2/3 of cases); in contrast, methamphetamine was more commonly used in HIV-positive individuals (Blomquist et al. 2020). Among MSM, illicit drugs used during chemsex practice vary substantially across European cities (Schmidt et al. 2016). In Australian, South-East Asian and North American contexts, to date, mephedrone use appears rare, and use of GHB remains at low levels (Bourne et al. 2018). During the descent phase, which may last up to 3 days with cathinones and, up to 5 days with metamphetamine, benzodiazepines or alcohol are often used.

### Prevalence and characteristics of chemsex use

Chemsex practice has been mostly studied in MSM (Malandain et al. 2020). This may reflect increased funding for AIDS research in MSM as compared to that for paraphilic disorders or CSBD per se. Prevalence estimates of chemsex in MSM range widely from 6% to 45% in the previous 6 or 12 months (Edmundson et al. 2018; for review see Íncera-Fernández et al. 2021). Edmundson et al. (2018) concluded that the prevalence estimate variations partly reflected differences in definitions used, substances identified, and populations assessed.

Chemsex prevalence was estimated at 22.5% in a French population of university students (166 males and 512 females), and alcohol was mainly used, but the use of illicit drugs in sexual contexts gradually increased in the general population. Self-reported sexual orientation of individuals engaging in chemsex was as follows: 68.6% heterosexual and 31.3% homo/bisexual as compared with 80.9% and 19% in students who did not report chemsex, respectively (Malandain et al. 2022). The age of individuals engaging in chemsex ranged between 30 and 44 years in the populations studied.

Living with HIV has been linked to chemsex in many studies (for review see Íncera-Fernández et al. 2021). Individuals with HIV who engage in chemsex are typically slightly older than those who engage without HIV (Hibbert et al. 2019; Blomquist et al. 2020). Regarding sexual practices, two-thirds of HIV-positive individuals reported any anal intercourse with casual partners; of these men, three-quarters reported one or more condomless acts in the previous year. HIV-infected MSM who use crystal meth were more likely to engage in unprotected anal intercourse, have multiple sex partners and group sex, find sexual partners on the internet, have sex with people who inject drugs, and be intoxicated during sex, compared to MSM who did not use crystal meth, regardless of their HIV status, as reviewed in (Rajasingham et al. 2012). McCormack et al. (2016) reported that almost half of individuals using pre-exposure prophylaxis (PrEP) had used methamphetamine, GHB/GBL or mephedrone in the past 90 days. Yet, in another study, Sewell et al. (2017) reported that the level of PrEP use and post-exposure prophylaxis (PEP) use was 4.5% and 14%, respectively, in a population using chemsex.

The prevalence of intravenous use of illicit drugs for sexual purposes is between 1% and 50%; among people engaging in slamsex, 5–56% shared injecting equipment (Edmundson et al. 2018; Íncera-Fernández et al. 2021). Moreover, up to a quarter of MSM attending sex parties, especially those who used methamphetamine (up to 76% in this subgroup), also consumed erectile enhancement drugs. Phosphodiesterase-5 inhibitor use (sildenafil, tadalafil and vardenafil) in combination with chemsex drugs is frequent in MSM and in people living with HIV (for review see Giorgetti et al. 2017). This simultaneous use is associated with cardiotoxicity and an increased risk of death. Some of the chemsex drugs used may also interact with protease-inhibitor treatments in HIV-positive patients.

### Adverse effects and psychiatric symptoms associated with chemsex

Both metamphetamine and mephedrone are associated with high levels of sexual pleasure and especially disinhibition, which is what many individuals engaging in chemsex seek. However, chemsex may be associated with negative consequences such as depression, paranoia and other psychotic symptoms, anxiety disorders, dependence, financial difficulties, emotional trauma or sexual violence or abuse. Chemsex has been identified as a risk factor for transmission of HIV, hepatitis C, and other sexually transmitted infections (STIs). This risk is enhanced given that individuals engaging in chemsex often have multiple sex partners (>10 in the past 3 months; 2.1 times higher adjusted odds as compared to MSM who do not report chemsex). In addition, individuals engaging in chemsex often have at-risk sexual practices such as condomless anal sex with a partner of unknown HIV status or who is HIV-positive or sharing equipment when injecting drugs (Blomquist et al. 2020).

Chemsex is associated with significant morbidity and mortality. In London, a gay man dies approximately every month in chemsex contexts. In 2018 in Lyon (France), 20 chemsex-related deaths were reported (CoreHIV 2018). Between 1995 and 2013, 21 GHB-/GBL-associated deaths were reported in the literature. In particular, significant caution is necessary when ingesting GHB/GBL with alcohol, benzodiazepines, phosphodiesterase-5 inhibitors, stimulants and ketamine (Corkery et al. 2018). In addition to the risk of death, a direct neurotoxic effect was reported with methamphetamine. Finally, chemsex-related crimes have also increased (Strudwick 2022).

Chemsex has been linked to poor quality of life. Fourteen to 25% of individuals engaging in chemsex have experienced negative impacts on their psychosocial functioning (Maxwell et al. 2019). Twenty-five percent of 209 MSM practicing chemsex expressed a need for professional counselling on chemsex-related issues (Evers et al. 2020).

MSM practicing chemsex were more likely to experience depression, anxiety or SUDs, especially those who have used intravenous drugs during slamsex (Íncera-Fernández et al. 2021). Prevalence of anxiety (17.9%) and depression (28.3%) was also high in an Australian cohort of 3017 gay and bisexual men (Prestage et al. 2018). Bui et al. (2018) reported associations between slamsex and depression (55.8%) and anxiety (42.5%).

Bourne et al. (2015) reported that among MSM engaging in chemsex, 10% reported to have overdosed on GHB with loss of consciousness (also called ‘G hole’) and therefore could not have given consent to the sexual acts that occurred but still did not refer to it as sexual assault or rape (Bourne et al. 2015). Drückler et al. (2021) have observed that 8.8% of individuals engaging in chemsex reported loss of consciousness due to illicit drugs and/or alcohol use; 21.2% engaging and 16.7% not engaging in chemsex reported any non-consensual sexual experience in the past 5 years (Drückler et al. 2018). Indeed, research indicates that chemsex increases the risk for non-consensual sexual acts, rectal trauma or penile abrasions (Bourne et al. 2015). Bohn et al. (2020) reported adverse consequences such as dyscontrol regarding loss of time and money spent for chemsex activities or number of substances used on one occasion (49.6%), negative impact on social functioning (33.6%), psychotic symptoms (13.2%), and physically aggressive behaviour towards others (2.9%).

Donnadieu-Rigole et al. (2020) reviewed studies reporting adverse effects associated with illicit drug use in the context of chemsex. Psychiatric symptoms such as anxiety, depression, paranoia, and confusion were reported with cathinone and methamphetamine use, and suicidal ideation, agitation and violence were reported with cathinones. Psychotic symptoms and alterations of executive and memory functions were observed with methamphetamine. Coma with hospitalisation in intensive care unit was observed with GHB/GBL (G hole). Dependence may be associated with all compounds. Cardiac ischaemia and hypertension may be associated with both cathinones and methamphetamine. Pulmonary arterial hypertension, heart rhythm disturbances and cardiomyopathy may be associated with methamphetamine. Sympathomimetic syndrome and rhabdomyolysis, which may require intensive care may be associated with cathinones. Finally, dental and periodontal diseases have been reported with methamphetamine use.

### Comorbidity with CSB or addictive disorders

Chemsex has close relationships with CSB and addictive disorders. Yet, few studies have reported the prevalence of co-occurring addictive disorders or CSB in individuals engaging in chemsex.

Associations between chemsex, alcohol use disorder and tobacco use has been reported (Edmundson et al. 2018). Sewell et al. (2017) found a high level of alcohol consumption in 12.9% of MSM. Brogan et al. (2019) reported a prevalence of alcohol dependency of 20.1% in 5165 MSM in Canada. In Brazil, among 1048 MSM, the prevalence of alcohol and illicit drug use in the previous 3 months was 89% and 49%, respectively, and 27% reported the use of alcohol during chemsex (Torres et al. 2020). Prevalence of alcohol (20.4% reported high levels of alcohol use) and illicit drug use (52.2%) was high in an Australian cohort of 3017 gay and bisexual men (Prestage et al. 2018). Among people recently using methamphetamine (11.2%), 28.0% were dependent. Schmidt et al. (2016) analysed data from 55,446 MSM living in 44 European cities and reported that alcohol and tobacco were by far the most commonly used drugs, whereas chemsex practice was reported in 0.4% (in Sofia) up to 16.3% (in Brighton, UK) of MSM. Unfortunately, the relationship between both tobacco and alcohol use and chemsex was not analysed. Yet, these authors recommended that MSM reporting chemsex use receive brief intervention for alcohol and SUDs in addition to HIV prevention strategies.

In summary, most studies conducted in people engaging in chemsex have focussed on HIV risk and sometimes on alcohol or illicit drug use in MSM but not on dependence/SUDs and co-occurring behavioural addictions or CSB. Surprisingly, the prevalence of CSB, which is often observed in cinical practice in people engaging in chemsex, has been rarely studied. Recently, a prevalence of 23% of CSB in 341 gay, bisexual and other MSM (38% were using illicit drugs) was reported using the Sexual Compulsivity Scale (Achterbergh et al. 2020).

## Correlates of CSBD

The essence of human sexual behaviour remains complex, and little is known about the brain pathways that regulate sexual behaviours. Many addiction-related brain networks have been revealed through the identification of neural circuits involving the regulation of reward processes using animal models (Nutt et al. 2015). Involving mesolimbic incentive salience circuitry, reward networks have been implicated in behaviours in which motivation has a central role, including eating and having sex.

### Biological correlates

Key neurochemical and hormonal substances that may influence sexual behaviour are monoamines such as dopamine or serotonin and cortisol and testosterone (Chatzittofis et al. 2022). In general, dopamine and testosterone may be considered necessary for, rather than leading to increases in sexual behaviours, while serotonin may have rather sexually inhibiting effects. The role of cortisol is less clear so far. However, these statements are an oversimplification.

Dopamine has been proposed to be a key neurotransmitter for the development of addictive behaviours due to its actions within reward circuitry, although its centrality to or role in behavioural and drug addictions has been questioned (Potenza 2013, 2018; Nutt et al. 2015). In animal models, dopamine can facilitate both sexual desire and sexual intercourse, suggesting a possible role in CSB as well (Hull et al. 2004). Rewarding stimuli typically lead to a release of dopamine in the ventral tegmental area projecting to the nucleus accumbens, the prefrontal cortex and amygdala (Baik 2013). Thereby, the amygdala may allocate an emotional reaction to stimuli and modulate the motivational states of individuals, whereas the prefrontal cortex may have greater responsibility for cognitive appraisal and control of stimuli (Baik 2013). Suggestions that dopamine may contribute possibly to CSBD come from patients with Parkinson’s disease who are treated with dopamine replacement therapy which has been associatied with CSB (Klos et al. 2005; Moore et al. 2014). However, as described above, the aetiology of CSB and/or behavioural addictions in Parkinson’s disease appears multifactorial, and the extent to which dopamine-related findings in Parkinson’s disease (a condition characterised by marked dopamine-related pathology) extend to other populations warrants direct examination. Neuromodulators (e.g., opioids) may influence the amount of dopamine released in response to stimuli, with the salience in part related to the intensities of dopamine pulses when individuals are exposed to rewarding stimuli (Nummenmaa et al. 2018). The underlying mechanisms may involve a decrease in the release of GABA when opioids bind to receptors on interneurons. As the suppressive function of the interneuron is obstructed, an increased dopamine signalling from the ventral tegmental area to nucleus accumbens may occur. In addition to the μ and δ opioid receptors located on GABAergic neurons in the ventral tegmental area, there are also opioid receptors in the nucleus accumbens itself that may directly influence dopamine release. In sexual contexts, opioids may contribute to the euphoria of orgasm, and eventually lead to a refractory period with impaired sexual functioning. Similarly, the accumulation of opioids (e.g., often seen with chronic opioid use) may produce erectile dysfunction, less sexual desire, and delayed ejaculation (Sathe et al. 2001).

Serotonin has mainly inhibiting effects on sexuality. The binding of serotonin to its 5-HT_2C_, 5-HT_1B_, and 5-HT_1C_ receptors may inhibit sexual responsiveness (Pfaus 2009; Köhler et al. 2016; Pehrson et al. 2016). In male rats, infusing serotonin into the hypothalamus or nucleus accumbens can generate delayed ejaculation (Fernández-Guasti et al. 1992). Increased levels of serotonin in the hypothalamus may inhibit sexual motivation and testosterone signals, while increased levels of serotonin in the prefrontal cortex may enhance emotional resilience and impulse control (Leeman and Potenza 2013). Consequently, systematic reviews and meta-analyses suggest that up to 80% of individuals being treated with SSRIs experience some reduced sexual desire and functioning (Serretti and Chiesa 2009). Furthermore, previous research has found less activation in the right postcentral gyrus, left superior frontal gyrus and pons in depressive individuals being treated with SSRIs while viewing sexual images, leading the authors to conclude that sexual dysfunctions during SSRI treatment might be due to less activation in these brain areas (Kim et al. 2009). In a pharmacological challenge study, the administration of the SSRI paroxetine over 7 days led to attenuated neural activations in different brain regions involved both in sexual interest and a sexual arousal network in heterosexual men from the general population when passively viewing erotic video clips (Abler et al. 2011). However, studies explicitly examining the role of dopamine and serotonin in individuals with CSBD are to our knowledge missing so far.

Especially in men, testosterone has mainly sexually stimulating effects, and this hormone is a prerequisite for sexual desire and responsiveness. Studies with men who have been convicted for or who are at risk of commiting a sexual offence and who have been treated with testosterone-lowering medications have repeatedly shown that low testosterone levels are associated with decreased sexual desire, sexual functioning and CSB (Turner et al. 2013; Turner and Briken 2018; Landgren et al. 2021). In contrast, testosterone supplementation leads to improved erectile function and higher sexual desire compared to placebo in men with erectile dysfunction. Moreover, patients with lower testosterone levels at baseline reported the most benefit from testosterone supplementation (Corona et al. 2017). In postmenopausal women, testosterone administration also led to increased sexual desire, responsiveness, and arousal (Islam et al. 2019). However, this does not necessarily mean that above-average testosterone levels are associated with increased sexual desire or frequency of sexual behaviours up to a level that they can be considered as CSB. For example, no differences in morning testosterone plasma levels were found between 67 men with CSB and 39 age-matched men without CSB (Chatzittofis et al. 2020). In that study, men with CSB had, however, higher luteinizing-hormone plasma levels than non-CSB men.

In many psychiatric disorders, including substance and behavioural addictions (Geisel et al. 2015; Kaess et al. 2017), dysregulation of the hypothalamic-pituitary-adrenal (HPA) axis has been observed (e.g., Fernandez-Guasti et al. 2012; Vinson and Brennan 2013). One previous study found that cortisol might facilitate sexual arousal, while sexual arousal itself did not change cortisol levels (Goldey and van Anders 2012). However, other studies found the opposite (Rowland et al. 1987; Exton et al. 2000). In a more recent study with 67 men with CSBD who were compared to 39 healthy controls, a higher rate of non-suppression in the dexamethasone suppression test and higher ACTH levels after dexamethasone administration were found among men with CSBD, pointing towards a hyperactive HPA axis in men with CSBD (Chatzittofis et al. 2016). Baseline cortisol levels also negatively correlated with CSB severity (Chatzittofis et al. 2016). In a genome-wide methylation pattern analysis on the above sample, four CpG sites associated with the corticotropin releasing hormone (CRH) gene, the corticotropin releasing hormone receptor 2 gene and the glucocorticoid receptor gene were hypomethylated in men with CSBD (Jokinen et al. 2017), pointing towards epigenetic changes in HPA-axis-related genes in men with CSBD.

It has been suggested that oxytocin may be implicated in CSB due to relationships with sexual behaviour, stress regulation, reward processing and bonding (Burri et al. 2008; Heinrichs et al. 2009). Oxytocin largely has inhibitory effects on addictive behaviours (Sundar et al. 2021). Men with CSB have demonstrated higher oxytocin plasma levels compared with non-CSB men (Flanagan et al. 2022). Furthermore, oxytocin levels were positively related to CSB symptoms, and a CSB-specific cognitive behavioural treatment program led to a significant reduction in oxytocin plasma concentrations (Flanagan et al. 2022). However, a study of men with problematic pornography use (PPU) investigating oxytocin and the related hormone arginine-vasopressin (implicated in generating aggressive and vigilant states in social-affiliative situations) demonstrated different findings (Kor et al. 2022). Specifically, higher levels of arginine-vasopressin and arginine-vasopressin dominance were observed in men with PPU versus those without. Men with PPU demonstrated greater oxytocin increases when viewing videoclips of neutral/positive social interactions. Diminished empathy and greater psychopathology were observed in men with versus without PPU, with a structural equation model suggesting 3 paths leading to pornography-related CSB: (1) direct path from psychiatric symptoms (strongest correlation); (2) direct path from arginine-vasopressin levels; and (3) indirect path from oxytocin being positively related to empathy and empathy being inversely related to pornography-related CSB. As such, oxytocin may have different relationships with CSB based on the type of CSB, although this notion is currently speculative.

### Brain imaging

#### Structural brain measures

Studies of structural brain measures related to CSBD are few, as reviewed and described elsewhere (Kühn and Gallinat 2016; Kowalewska et al. 2018; Kuiper and Coolen 2018; Stark et al. 2018).

Miner et al. (2009) explored white matter microstructure in eight men with CSBD and eight men without. Diffusion-tensor-imaging (DTI) data were collected, and mean diffusivity and fractional anisotropy in inferior and superior frontal areas were compared. Although the authors expected to find poorer white matter integrity in the frontal lobes of participants with CSBD, there were no significant differences between the clinical sample and the control group. However, lower mean diffusivity in superior frontal regions in males with CSBD was observed.

Schmidt et al. (2017) examined gray-matter volume (GMV) in 92 individuals (23 men with CSBD and 69 men without). They found larger left amygdala GMVs in participants with CSBD. Kühn and Gallinat (2014) used voxel-based morphometry to study a subclinical sample of 64 men. They observed a negative association between GMV in the right caudate and self-reported pornography use (hours/week). Seok and Sohn (2018) explored whether individuals with CSBD showed altered GMV in the temporal lobe given its role in the regulation of human sexual arousal (Baird et al. 2007). The authors found smaller GMVs in the right middle temporal gyrus and left superior temporal gyrus in the 17 individuals with CSBD, compared to the 17 without. A more recent study examined men with CSBD (*n* = 26), gambling disorder (*n* = 26), alcohol use disorder (*n* = 21) or no condition (*n* = 21) (Draps et al. 2020). Affected individuals as a group showed smaller frontal pole volumes in the orbitofrontal cortex, although these differences seemed less pronounced in the CSBD group. An inverse relationship between CSBD symptom severity and GMVs in the anterior cingulate was observed. As studies conducted to date have been limited, findings should be interpreted cautiously. Future studies, particularly those employing larger samples and using longitudinal designs, are needed (Stark et al. 2018). In addition, a past history of sexual abuse, which was not assessed/reported in most studies, may be a potential bias.

#### Functional studies

Functional magnetic resonance imaging (fMRI) has been used to investigate CSBD (Kuiper and Coolen 2018). Task-based research has enabled the exploration of functional connectivity and regional activation (Kowalewska et al. 2018). In addition, most studies involving sexually explicit materials have focussed on cue-induced reactivity (Kraus et al. 2016b).

##### Studies with healthy participants

Studies using healthy participants have shown that exposure to sexually explicit content increases signals in regions associated with reward and motivation, such as the amygdala, ventral striatum and anterior cingulate cortex (Strahler et al. 2018). Kühn and Gallinat (2014) observed a negative correlation between weekly hours of pornography use and resting-state functional connectivity between the left dorsolateral prefrontal cortex and the right caudate. The authors suggested a role for enhanced habituation due to an intense stimulation of the reward system.

##### Studies of participants with CSB/CSBD

Electroencephalography (EEG) was used to study 122 men and women (Prause et al. 2015), some of whom exhibited PPU, which may be considered a form of CSBD, although alternate conceptualizations have been considered (Brand, Rumpf, King, et al. 2020). In an event-related potential design, participants with PPU, compared to those without, showed lower late positive potentials.

Functional MRI studies of men with CSBD have shown an association between enhanced sexual desire when exposed to sexually explicit content and enhanced blood oxygenation level dependent (BOLD) signal in the dorsolateral prefrontal cortex, dorsal anterior cingulate gyrus, caudate nucleus and thalamus (Seok and Sohn 2015). Banca et al. (2016) reported that the dorsal anterior cingulate cortex habituated more quickly in response to sexual cues in 22 CSBD men as compared to 40 men without CSBD. This region may be important in reward expectation. Similar studies have also highlighted enhanced activity in the amygdala, dorsal anterior cingulate cortex and ventral striatum during the anticipation of erotic stimuli (Voon et al. 2014; Gola and Draps 2018), in men with CSBD/PPU as compared to those without. Further, connectivity between the amygdala, dorsal anterior cingulate cortex and ventral striatum was linked more strongly to sexual desires in men with CSBD as compared to those without (Voon et al. 2014). Schmidt et al. (2017) reported reduced resting-state functional connectivity between the left amygdala and bilateral dorsolateral prefrontal cortex in participants with CSBD compared to controls. Klucken et al. (2016) observed increased amygdala activation with sexual cues in men with CSBD and men with CSBD demonstrated decreased connectivity between the prefrontal cortex and the ventral striatum. These findings suggest that individuals with CSBD may exhibit less prefrontal regulatory control over emotional and motivational processes. Seok and Sohn (2018) found altered resting-state functional connectivity between the left superior temporal gyrus and right caudate in individuals with CSBD after controlling for sexual activity. Furthermore, the functional connectivity was linked to the severity of CSBD. Using a modified monetary incentive delay task involving sexual and monetary cues and rewards, Gola et al. (2017) found that men with PPU, compared to those without, showed greater ventral striatal activation specifically to cues signalling erotic trials. Furthermore, the degree of ventral striatal activation was associated with response times during erotic trials, sexual addiction severity, amount of pornography consumed and weekly masturbations.

Existing neuroimaging studies suggest possible neurobiological mechanisms underlying CSBD. Regions implicated include the amygdala, thalamus, striatum and dorsal anterior cingulate and prefrontal cortex during processing of sexually explicit materials (Kuiper and Coolen 2018). However, studies are few and have multiple limitations, such as the predominant use of cross-sectional designs, exclusive use of men in most studies, and small sample sizes (Kraus et al. 2016a). Additional research is needed to understand associations between neurobiological, neurocognitive, and clinical aspects of CSBD. Investigations of cognitive processes suggest attentional biases (Mechelmans et al. 2014) and approach tendencies (Snagowski and Brand 2015; Sklenarik et al. 2019, 2020) similar to those observed in substance addictions (Kraus et al. 2016a), and these should be investigated further to understand better the neural and clinical correlates. Further, the aetiology of brain-behaviour relationships should be examined, including longitudinal, developmental studies. Integrating neuroimaging findings with other measures, such as genetics and epigenetics, and considering transdiagnostic factors in the process, should be informative (Kowalewska et al. 2018). Integrating brain imaging measures into treatment trials should provide insight into neural features associated with recovery and brain features linked to active ingredients of behavioural therapies and pharmacotherapies, as is being done with other psychiatric conditions (Balodis et al. 2016; Zhang et al. 2016a, 2016b; Garrison et al. 2017). Identifying individual differences with clinical relevance may help advance precision-medicine approaches.

### Psychosocial correlates

Despite CSBD only being recently recognised by the WHO, CSB has been extensively studied in psychiatric, psychological, and social science research over the past three decades. A recent review concluded that, since 1995, over 400 empirical studies of CSB had been published (Grubbs, Hoagland, et al. 2020). Importantly, much of this research has focussed on the extent to which CSBs are associated with or predicted by a variety of psychological covariates and individual differences. For example, some work suggests that gay and bisexual men are more likely to report distress over self-perceived CSBs (Dickenson et al. 2018) in comparison with heterosexual men and women and gay and bisexual women. Similarly, men in general are more likely to report elevated levels of CSBs in comparison with women (Grubbs et al. 2019). However, beyond demographic correlates alone, there are multiple psychosocial correlates of CSBs that should be considered. Below, we consider some general findings related to these psychosocial correlates.

#### Personality features

A number of studies have demonstrated that CSB is often robustly associated with a variety of personality features. Of the personality features most commonly associated with symptoms of CSB, impulsivity is a frequent covariate of such behaviours (Reid, Bramen, et al. 2014; Reid, Cyders, et al. 2014; Reid et al. 2015; Engel, Kessler, et al. 2019; Bőthe, Tóth-Király, et al. 2019). More specifically, higher levels of impulsive tendencies seem to be consistently associated with and predictive of self-reported problems with CSB. These findings are evident in both community and clinical samples.

Additionally, CSB has been associated with higher levels of emotional dysregulation, neuroticism, and negative affectivity (Reid, Bramen, et al. 2014; Walton et al. 2017). In general, greater proneness towards the experience of negative affect seems to be associated with greater engagement in CSBs. Prior conceptions of CSB (i.e., the DSM-5 proposal for hypersexual disorder) and many assessments of CSB (e.g., the HBI-19; the Compulsive Sexual Behaviour Inventory) have included the use of sexual behaviour to cope with negative mood, stress, or worry as a symptom of CSB (Gola et al. 2020). Although the diagnostic criteria for CSBD do not include such motivations for the disorder, there is a rich literature that suggests that CSBs are indeed associated with higher levels of negative affect in general and a desire to use sexual behaviour to cope with such affect.

#### Morality/religion

Religiousness and conservative morality might be important psychosocial covariates of CSBs (Grubbs and Perry 2019; Lewczuk et al. 2020; Grubbs, Kraus, et al. 2020; Briken et al. 2022). Specifically, there are a number of studies that now show that moral disapproval of sexual behaviours such as pornography use often may predict self-reported addiction to or dysregulation in those behaviours (Grubbs et al. 2019; Walton 2019; Lewczuk et al. 2020). That is, to the extent that people find certain sexual behaviours to be morally wrong or to violate their personal or religious beliefs, it seems that those people are more likely to interpret any engagement in those behaviours as indicative of a behavioural addiction (Lewczuk et al. 2020; Grubbs, Kraus, et al. 2020). This tendency has been labelled *moral incongruence* in prior literature. Case studies (Kraus and Sweeney 2019) and retrospective chart reviews in treatment settings (Cantor et al. 2013) suggest that moral incongruence might lead some people to seek treatment for CSBs when they are actually just dealing with extreme moral distress over normal or even low levels of sexual behaviour. The diagnostic criteria for CSBD note that distress about sexual behaviours that exclusively arises from religious or moral concerns is not enough to justify a diagnosis of CSBD (Kraus et al. 2018). Therefore, clinicians and mental health professionals should not consider moral incongruence in diagnosing CSBD. However, prior research of marriage and family therapists (Hecker et al. 1995) and social workers (Droubay and Butters 2020) has found that more religious clinicians are more likely to diagnose clients with sexual addiction or describe sexual behaviours as addictions. Accordingly, in any treatment or research setting, it is important that professionals helping people with CSB are aware of the religious and cultural contexts (including moral beliefs) of their patients and themselves. The extent to which moral incongruence applies to other addictive behaviours also warrants consideration as data suggest that individuals with multiple behavioural addictions exhibit moral incongruence towards their behaviours (Lewczuk et al. 2021). Furthermore, most studies of moral incongruence in CSBD do not consider the content of the sexual behaviour (e.g., whether the content of the pornography viewed compulsively is focussed on violent or rape behaviours (Gola et al. 2020)). As many people with drug addictions find that their behaviours run contrary to their moral belief system and for other reasons, the extent to which moral incongruence represents a separate path towards perceived addiction has been questioned (Brand et al. 2019).

In summary, the current literature suggests that the psychosocial and cultural contexts of an individual are important in understanding the presentation and experience of CSB symptoms. CSBs may be predicted by personality and individual differences such as greater propensity towards negative affect and higher impulsivity. Similarly, psychiatric disorders such as ADHD also seem to enhance the risk of experiencing CSB, which suggests that future research may be well-served by attempting to identify clear individual risk factors for the future development of CSBD. Finally, CSBs often co-occur with a range of other impulsive and addictive behaviours such as substance use, problematic gaming, and problematic gambling. Such comorbidity highlights the need to assess for CSBD in addiction treatment settings, particularly in those where other behavioural addictions may already be a focus of treatment.

## Assessment of CSBD

Numerous questionnaires and interviews can be used to assess CSB and can assist in deciding whether a certain individual should be diagnosed with CSBD. At this point, we do not want to give a full overview of all existing instruments as there exist recent reviews (Womack et al. 2013; Turner, Schöttle, et al. 2014; Montgomery-Graham 2017). However, there are some scales that can be recommended due to their good reliability and validity indices and wide distribution.

### Self-report scales

#### Hypersexual Behaviour Inventory (HBI-19; Reid, Garos, et al. 2011)

The HBI-19 (Reid, Garos, et al. 2011) is useful for assessing CSB psychometrically (Womack et al. 2013; Turner, Schöttle, et al. 2014; Montgomery-Graham 2017). The HBI-19 is a well validated scale and relatively brief. However, it is constructed for the assessment of hypersexual disorder symptomatology as proposed but not included into the DSM-5; it does not reflect the different diagnostic guidelines for the ICD-11 diagnosis of CSBD.

The HBI-19 consists of 19 items, which are divided into three subscales: control, coping, and consequences. The Control scale assesses tendencies to stop sexual behaviour, the Coping scale assesses whether sexual behaviour is used to manage stress, and the Consequences scale assesses whether sexual behaviour interferes with the achievement of personal goals. A score above 53 points (range: 19–95 points) suggests the presence of clinically relevant CSB with respect to hypersexual disorder as proposed by Kafka (2010). There exist translations into German (Klein et al. 2014), Spanish (Ballester-Arnal et al. 2019), Italian (Ciocca et al. 2020), and Hungarian (Bőthe, Kovác, et al. 2019).

#### Sexual Compulsivity Scale (SCS; Kalichman et al. 1994)

The SCS is the oldest among recommended self-report scales. It consists of 10 items that can be answered on a four-point scale ranging from ‘not at all like me’ to ‘very much like me’. The SCS was developed to assess sexual risk behaviour in MSM; however, in the initial validation study, no association with sexual risk behaviour was observed (Kalichman et al. 1994). The SCS measures the impact of sexual thoughts on daily functioning and difficulties controlling sexual thoughts and behaviours, though it is not a clinically validated instrument.

#### Hypersexual Behaviour Consequences Scale (HBCS; Reid, Garos, et al. 2012)

According to the developers, it was their aim to create a scale, ‘that could (1) be used in clinical populations seeking help for hypersexual behaviour, (2) provide greater specificity in the identification of consequences encountered by respondents, (3) discriminate between consequences incurred by individuals who engage in solo vs. relational sexual behaviour […]’ (Reid, Garos, et al. 2012, p. 116). The final scale consisted of 22 items that can be answered on a 5-point scale ranging from ‘hasn’t happened and is unlikely to happen’ to ‘has happened several times.’ Higher scores reflect greater emotional dysregulation and impulsivity and lower levels of life satisfaction.

#### Compulsive Sexual Behaviour Disorder Scale (CSBD-19; Bőthe et al. 2020)

In contrast to the HBI-19, the CSBD-19 is the first scale that captures CSB based on the diagnostic criteria for CSBD in the ICD-11 (Bőthe et al. 2020). It consists of 19 items that can be grouped into five subscales: Control over sexually compulsive behaviour, Involvement (i.e., to what extent does sexually compulsive behaviour represent a central focus in the individual’s life), Recidivism (i.e., unsuccessful attempts to reduce the frequency of sexually compulsive behaviour), Distress, and Negative Consequences. A score of 50 or higher (range 19–76) indicates a high risk for the presence of CSBD according to the ICD-11 definition.

#### The Compulsive Sexual Behaviour Inventory (CSBI; Miner et al. 2007)

Participants rate each of the 13 items on a 5-point Likert scale ranging from 1 (never) to 5 (very frequently). A total score of 35 or greater has been shown to be a sensitive and specific cut-off for distinguishing individuals who meet criteria for a CSB clinical syndrome and is accurate 79% of the time. A score of 35 or higher indicates a high probability of meeting diagnostic criteria and warrants further evaluation to ascertain the diagnosis of CSBD.

#### Sexual Addiction Screening Test (SAST, Carnes 1983)

The SAST consists of 25 items and assesses the presence of addictive sexual behaviours and symptoms. It comprises three subscales: sexual preoccupation, signs and symptoms of impaired control, and problems resulting from sexual behaviours. Each item has to be answered dichotomously with either yes or no. Psychometric properties can be considered as good to excellent.

#### PATHOS (Carnes et al. 2012)

Participants answer yes or no to each of the six questions. A final score >3 reflects sexual addiction. PATHOS is a brief and easy to use self-report screening questionnaire for sexual addiction. It was extracted from the Sexual Addiction Screening Test (a 25-item measure for symptoms of sexual addiction from Carnes, 1989). This scale is easily used to identify participants with potential sexual addiction who would warrant additional assessment. This scale awaits psychometric validation. There is also a French translation that has yet to be validated (Acuna Vargas S. and Karila L. et al. personal communication; unreferenced).

#### The Pornography Craving Questionnaire (PCQ; Kraus and Rosenberg 2014)

Participants rate each of the 12 items on a 7-point Likert scale ranging from 1 (disagree completely) to 7 (agree completely). Higher scores signify a greater craving for pornography. A final score ≥5 reflects greater craving for pornography. The psychometric properties of the PCQ range from good to excellent, and it has good convergent, criterion, discriminant, and predictive validity of pornography use (Kraus and Rosenberg 2014).

#### The Problematic Pornography Use Scale (PPUS; Kor et al. 2014)

The PPUS measures problematic pornography use on fou domains: distress and functional problems, excessive use, control difficulties, and use for escape/avoid negative emotions. It consists of 12 items which can be answered on a 6-point scale ranging from 0 (never true) to 5 (almost always true). Higher scores represent higher levels of problematic pornography use. The PPUS demonstrated good internal consistency.

#### The Problematic Pornography Consumption Scale (PPCS-18; Bőthe, Tóth-Király, et al. 2018)

The PPCS consists of 18 items, which can be gouped into six subscales: salience, mood modification, conflict, tolerance, relapse, and withdrawal. All items have to be answered on a 7-point scale ranging from 1 (never) to 7 (all the time). The PPCS-18 has shown excellent internal consistency.

### Clinical interviews

#### Hypersexual Disorder Screening Inventory (HDSI; Reid, Garos, et al. 2012)

The HDSI was developed to be used in the DSM-5 field trials to screen for hypersexual disorder based on the criteria suggested for inclusion in the DSM-5. The HDSI consists of seven items, which can be answered on a four-point scale ranging from never true to almost always true. While the first five items assess the intensity of sexual fantasies, urges and behaviours, the last two items refer to personal distress or social impairments. It can be administered as an interview or as a self-report scale. Endorsing at least four items in the first section and one item in the second section qualifies for a positive screen for CSBD. Among currently existing instruments, the HDSI has been described as being particularly psychometrically sound (Montgomery-Graham 2017).

#### The Hypersexual Diagnostic Clinical Interview (HD-DCI; Reid et al. 2012)

The HD-DCI is a structured interview assessing each of the proposed DSM-5 criteria of hypersexual disorder. It was created based on traditional structured diagnostic interviews. In the DSM-5 field trials a high reliability and strong psychometric properties were found.

## Theories relevant to treatment approaches

Regarding the aetiology of CSBD, the state of knowledge is still very limited and its aetiology is discussed controversially (Briken and Turner 2022; Grubbs et al. 2020). As with other disorders, antecedents, correlates, and mediators should be distinguished. These can be summarised in more complex models, for all of which, however, empirical testing is still pending. In addition, the umbrella term CSBD may describe aetiologically very heterogeneous symptom constellations, which may be distinguishable in terms of gender (Kürbitz and Briken 2021). In turn, considerations of aetiology and theory may also influence therapeutic approaches, especially in everyday clinical practice.

With a biopsychosocial understanding, there may be individual differences linked to vulnerabilities for CSBD, including genetically/constitutionally (Chatzittofis et al. 2022), that may interact with distal experiences and social factors. With regard to antecedents, traumatic events in childhood, namely both experienced sexual abuse (Slavin et al. 2020) and psychological trauma (possibly especially by fathers; Kingston et al. 2017; Knight and Graham 2017), have considerable empirical support. With regard to social factors, aspects such as the availability of sexual material (e.g., early exposure to pornography via digital media), negative attitudes towards sexual behaviour and simultaneous high-frequency behaviour or strong urges are likely to be significant. The experience of moral incongruence that arises in this context may contribute to the distress people with CSBD experience. People with CSBD may use sexual behaviour as a coping strategy, e.g., in dealing with boredom, depression, low self esteem. This coping mechanism may become dysfunctional and contribute to distress or other difficulties (e.g., assaults towards others).

Based on Bancroft’s theory of the Dual Control Model (‘Out of Control Model’ of CSBD, Bancroft et al. 2009; Bancroft and Janssen 2000) and Perelman’s Sexual Tipping Point Model (Perelman 2009), Briken (2020) has developed an integrated model to assess and treat CSBD. This model hypothesises CSBD to be a disorder where the interplay between excitatory and inhibitory factors may be imbalanced (Rettenberger et al. 2016). The model arranges aetiological, correlating and mediating factors in the function of excitatory and inhibitory factors and thus creates approaches regarding how imbalances in relationships may be brought back into balance by therapeutic approaches, e.g., using naltrexone to decrease excitation and SSRI to increase inhibition, although each may operate through other mechanisms as well. Improving how to deal with stress and how to increase self esteem using cognitive behavioural therapy may also be important.

Such clinically informed models, which allow individualised therapeutic strategies to be developed, are likely to be particularly important because the assignment to disorder categories has not yet led to clear results. The scarcity of empirical data opens space for the proposition of many explanatory models. They are based on the models of: obsessive-compulsive disorder (OCD), impulsive-control disorders (ICDs) and addictive disorders. Each one has been developed based on assumptions about aetiological mechanisms and with the aim of proposing effective treatment (Kingston and Firestone 2008; Garcia and Thibaut 2010).

In the ‘Addiction Model’, sexual addiction appears to include the core elements of addictions: a craving state prior to behavioural engagement or a compulsive engagement; impaired control over behavioural engagement; continued behavioural engagement despite adverse consequences (Gold and Heffner 1998). Diagnostic criteria for SUDs include life interference, tolerance, withdrawal and repeated attempts to quit. The same descriptions apply to certain cases of human sexual and/or attachment relationships. Therefore, several authors have suggested considering CSB as an addictive disorder (Carnes 1983; Gold and Heffner 1998). In addition, comorbidity between CSB and other addictions is high. Thus, pharmacological treatments used for SUDs may have potential for treating non-substance addictive disorders.

The ‘ICD model’ was proposed by Barth and Kinder (1987) who introduced the compulsive-impulsive model, in which patients with a CSB present a failure to resist sexual impulses. Individuals may experience transient relief from negative emotional states and subsequent distress resulting from the sexual behaviour and, as such, would satisfy the DSM ‘not otherwise specified’ and ICD-11 criteria for an impulse-control disorder. According to this theory, the impulsive component (for example, pleasure, arousal or gratification) would be responsible for the initiation of the cycle and a compulsive component would be involved in the persistence of the behaviour (Dell’Osso et al. 2006). A shift from compulsive components associated with the dorsal striatum (caudate and putamen) to impulsive components of the ventral striatum (nucleus accumbens and ventral tegmental area) may be observed (Fontenelle et al. 2011). Finally, SSRIs, which increase serotonin levels, improve impulsivity in both ICDs and CSB, although SSRIs do not carry formal indications for these disorders.

The ‘OCD model’ was proposed by Coleman (1991). He used the term ‘compulsive sexual behaviour’, making a parallel between the phenomenology of OCD and CSB. Both conditions may improve with SSRI treatment. However, CSB often immediately reduces anxiety whereas in OCD, anxiety is increased by obsessive thoughts and often not fully decreased by compulsive behaviours. Moreover, patients with CSB report pleasure from sexual behaviours, which is typically not observed with OCD patients.

In summary, these theoretical models suggest that CSBD might be subdivided into two subgroups: those primarily presenting with increased anxiety and negative feelings perhaps mediated by increased amygdala reactivity and those with increased cue reactivity perhaps involving the ventral striatum. Those more prone to use sex as a way of coping with anxiety and negative feelings may be more likely to respond to SSRIs. Those more driven by cue reactivity due to increased ventral striatum reactivity may be more likely to respond to naltrexone (Savard 2021).

## Treatment of CSBD

### Psychological treatment of CSB

There is little high-quality research related to the psychotherapeutic treatment of CSBs (Grubbs, Hoagland, et al. 2020). Although several studies have examined psychoeducational interventions, acceptance and commitment therapy (ACT), and cognitive-behavioural interventions, none have demonstrated efficacy across multiple trials or in women. Since 1995, despite over 100 studies of CSB occurring in clinical or treatment-seeking settings, there have been only 12 treatment studies published that detail at least one form of psychological treatment for CSB (Grubbs, Hoagland, et al. 2020). Of these 12 studies, all have been conducted in samples primarily consisting of men (i.e., in all samples, at least 70% of participants were men) and almost all have been conducted in Western countries. Importantly, only two made use of randomised control designs (Crosby and Twohig 2016; Hallberg et al. 2019). Even so, despite this relative dearth of research related to CSB treatment, there are some interventions that show promise. Below, we review the current empirical literature on the psychological treatment of CSBD.

#### Psychoeducational interventions

Psychoeducation is used to improve knowledge of patients and their families regarding the disorder (e.g., aetiology, functioning, symptoms, risk factors and available treatments). In general, psychoeducational interventions are popular from a public health and a treatment perspective as they offer the opportunity to intervene broadly at relatively low costs. In the realm of CSBs, psychoeducation is often quite important as the risks of CSBs (i.e., STIs, legal complications, financial consequences) may be generally unknown by individuals with CSB. Furthermore, psychoeducational interventions are of particular interest because of their relatively low cost and the ease with which they may be disseminated to populations in need (Zhao et al. 2015). Psychoeducational interventions are, typically, more cost-effective than intensive therapeutic protocols and may be disseminated easily in group settings or even via the internet (Poole et al. 2012; Grey et al. 2013). For various mental health concerns, psychoeducational interventions are effective at reducing problematic behaviours and decreasing future need for treatment resources (Shimodera et al. 2012).

At the time of the writing of these guidelines, two studies evaluated the efficacy of psychoeducational interventions for CSB. The first of these studies details an online psychoeducation intervention program called the Candeo Online Recovery Program for Problematic Pornography Use (Hardy et al. 2010). The Candeo program purports to deliver, via the internet, ten self-paced psychoeducational modules based on a cognitive behavioural therapy approach to mental health and an understanding of PPU as being related to both addiction and compulsive dysfunction (Hardy et al. 2010). Importantly, the full details of these modules or the psychoeducational interventions used are not publicly available as the program is not offered free of cost. Based on cross-sectional and retrospective data in a sample of 138 participants (97% men), Hardy and colleagues concluded that there was preliminary evidence of the efficacy of the Candeo program in reducing PPU. However, this initial study was conducted over a decade ago, and no further research has been published on this program.

The second study to make use of psychoeducational interventions details an in-person group-based approach to psychoeducation for CSB, entitled the Hall Recovery Course (Hall et al. 2020). This course purports to deliver a psychoeducational program via in-person workshop or residential groups (Hall et al. 2020). According to available descriptions of the program, the Hall Recovery Course incorporates aspects of various therapeutic frameworks (i.e., ACT, cognitive-behavioural therapy (CBT), relational psychotherapy, psychodynamic psychotherapy, and positive psychology) to provide participants in the program with greater knowledge of their personal problems and of CSB more broadly. Much like the Candeo program described above, this method also does not make materials for psychoeducation publicly available as the program is not offered free of cost.

In summary, only two psychoeducational interventions for CSB have been examined in the existing literature. Although both purport efficacy, neither makes their psychoeducational materials publicly available. Accordingly, at present, it is unclear whether or not psychoeducational interventions are efficacious in treating CSB. Even so, given the potential for psychoeducational programs to be widely disseminated as low-cost interventions, research in this area is needed and encouraged.

#### Cognitive behavioural interventions

Moving beyond more passive interventions (i.e., psychoeducation), there are a small number of studies examining more intensive therapeutic protocols. Two studies by the same research group have detailed primarily CBT interventions for CSB (Hallberg et al. 2017, 2019).

The available CBT approaches for treatment of CSB are comparable to CBT for addictive disorders (i.e., SUDs, gambling disorder) more broadly. The initial focus of CBT is to decrease addictive-like behaviours, change maladaptive core beliefs, and reduce perceived shame and stress and increase self esteem. In order to meet treatment goals, therapists confront irrational beliefs, stimulate problem-solving skills and provide lectures and homework. During CBT, therapists lead patients to focus on thoughts, feelings and behaviours triggered by their sexual urges. Therapists help patients to explore impulse control, triggers and negative-thinking patterns, aiming at behavioural change. Motivational interviewing (MI) techniques may be used to encourage change and establish a strong therapeutic alliance. Other techniques include self-monitoring through daily diaries and helping patients to gain consciousness of their thoughts, feelings and emotions of situations connected to maladaptive sexual behaviours (Rosenberger et al. 2011). Relapse prevention is also used in CBT to teach individuals who are trying to change behaviour how to anticipate and cope with situations that may lead them to relapse, how to build action plans and how to identify supportive people (Marlatt and Donovan 2005). As a self-control technique, relapse-prevention helps patients to develop skills to identify high-risk situations that may trigger relapse, change cognitive distortions or faulty thinking and cope with these situations, in particular how to deal with stress and how to get positive reward from other pleasurable activities.

Both of the above-noted studies (Hallberg et al. 2017, 2019) were concerned with the same intervention program, with the former study being a treatment feasibility study and the second being a randomised controlled trial of the intervention (the waiting list was used as a control group, an approach subject to bias given lack of complete blinding). In both cases, individuals meeting criteria for proposed DSM-5 diagnosis of hypersexual disorder participated in a 7-week psychotherapeutic group treatment using a CBT framework. Each week within this treatment program approached a different therapeutic goal (e.g., Week 1 dealt with basic introduction of cognitive-behavioural principles of behavioural change; Week 4 introduced behavioural-activation strategies; Week 6 introduced interpersonal behavioural activation and conflict management) via group work and individual homework and workbook completion. Across 137 participants that were predominately men, results of the study and longitudinal assessments at three and 6 months after treatment, the program demonstrated efficacy in reducing CSBs and in reducing impairment and distress related to CSBs. In seventy patients receiving CBT, there was a greater decrease in CSBD symptoms using the Hypersexual Current Assessment Scale as compared to 67 patients in the control group. Though preliminary and in need of independent replication, such findings suggest that CBT for CSB may be a viable, evidence-informed approach to treating CSB, particularly in men.

#### Acceptance and commitment therapy

Although more recently developed than CBT, ACT has a strong evidence basis for the treatment of multiple psychiatric disorders (Hayes et al. 2011; Arch et al. 2012). ACT is a transdiagnostic approach to treatment that seeks to address a variety of possible diagnoses by focussing on processes (i.e., psychological inflexibility) that may be common across multiple psychiatric conditions (Hayes et al. 2006).

Three published studies have examined the efficacy of ACT techniques for the treatment of PPU (Twohig and Crosby 2010; Crosby and Twohig 2016; Levin et al. 2017). Although CSBs subsume more behaviours than PPU only, data suggest that PPU is among the most common sexual behaviours reported in CSBD (Grubbs, Hoagland, et al. 2020). Accordingly, it is reasonable to examine protocols that treat PPU as relevant to the treatment of CSB more broadly.

Two published studies detailing ACT for PPU used individual therapeutic approaches (Twohig and Crosby 2010; Crosby and Twohig 2016). In both cases, men with self-reported PPU participated in an 8-session therapeutic protocol that focussed on basic tenets of ACT (e.g., psychological flexibility, cognitive defusion, acceptance, and committed action) as they applied to PPU. In both pilot studies and randomised trials, this approach was successful in reducing PPU and increasing self-reported efficacy to resist using pornography.

In a subsequent study examining ACT for PPU (Levin et al. 2017), a bibliotherapy approach was taken where participants were guided through a self-help style intervention that was based on the popular ACT book, *Get Out of Your Mind and Into Your Life* (Hayes 2005). Although the basic foci of this therapeutic intervention were the same as the above-mentioned studies, the delivery was entirely self-directed via the above book. Results of the study revealed that participants who completed the self-help book repored reductions in PPU. Several participants did not complete the book and did not report such reductions.

In sum, three studies provide preliminary evidence for the efficacy of ACT-based approaches to CSB. All three studies of this therapeutic approach were conducted exclusively with men and all three only focussed on PPU. As such, the general utility of ACT for the treatment of CSBs in women or CSBs that extend beyond PPU is not yet known. Even so, given the limited evidence for other interventions, ACT is currently one of the few evidence-based psychological treatments for CSB. Moreover, given that basic protocols for ACT are widely available and the approach’s purported utility transdiagnostically, it is likely that such an intervention could be easily disseminated to mental health providers if future evidence warrants such dissemination.

#### Family/couple therapy

Couples and family therapies have been reported as important methods to help rebuild trust and closeness, to improve communication skills, to advocate quality of time and to turn family member into allies during the recovery process (Hayden 2013). Partners and family members of patients with CSB may present sexual difficulties, distrust, betrayal, shame and negative self-esteem (Turner 2008; Kaplan and Krueger 2010). Some studies evaluating families of patients with CSB have emphasised the co-addictive role of spouses and family members (Schneider and Schneider 1996; Milrad 1999). Some authors have divided family therapy into four stages of recovery. In the first stage, or ‘pre-recovery stage’, spouses may reflect upon their fears that something is wrong and their ‘detective’ or snooping behaviours (e.g., checking wallet, cell phone, pockets and following the person). During this stage, spouses may confront their partners who typically deny their problems. In the second stage, known as the ‘crisis stage’, spouses often deal with depression, anxiety and low self-esteem secondary to the grief of the perception of having a wife or husband with CSBs. Throughout the next stage, called the ‘shock stage’, spouses often feel numb yet also optimistic about their partners’ recovery. Finally, in the ‘grief stage’, spouses often contemplate their losses and perspectives for the future. Milrad (1999) suggested that the stages may not follow a sequential order and that spouses may not experience all stages. Couples and family therapies may help partners to become aware of each other’s thoughts, perspectives, issues and struggles (Milrad 1999). Bird (2006) suggests that couples and family therapies may be more helpful than group or individual therapy for CSB. The main themes addressed in these therapies are typically establishing boundaries, restoration of trust, improving communication and intimacy (Milrad 1999; Bird 2006). Turner (2008) reported that intergenerational factors and cultural distortions are often important themes to address in therapy. Regarding individual therapy, different approaches for couples have been described (for review, Garcia et al. 2016). In a study performed with counsellors working with patients with CSB, the authors recommended the use of more than one treatment program during the therapeutic process (Swisher 1995). Other researchers (Milrad 1999; Schneider 2000) have suggested that individual therapy is necessary for patients with CSB before starting couples therapy. These authors have reported that CSB patients should address personal issues before exposing them to their spouses. Other authors have recommended that both partners attend self-help meetings. Schneider and Schneider (1996) have suggested that couples sex therapy may be incoporated in later stages of recovery. No randomised controlled trials of family/couples therapy for CSB have been published.

#### Self-help groups

Self-help group psychotherapy for the treatment of CSB has been adapted from the 12-step model and practice of Alcoholics Anonymous. Groups exist named ‘Sexual Anonymous’, ‘Sex and Love Addicts Anonymous’, ‘Sexaholics Anonymous’ and ‘S-Anon’ and ‘Co-dependents of Sex Addicts’ for patients and families, respectively. Some authors propose that self-help groups may be an adjunct to other treatments. Treatment goals are often focussed on helping the patient to stop or control their problematic sexual behaviour and to learn new coping strategies (Hardy et al. 2010). These meeting groups can facilitate recovery in individuals, helping them to become more honest with themselves and their family in a supportive atmosphere. These groups often provide a place for fellowship and support (Schneider and Schneider 1996). No results describing compliance or efficacy of self-help groups for CSB have been reported.

## Pharmacological treatment

The main aim of the present guidelines was to review the current state of pharmacological treatment in CSB and to provide recommendations concerning the use of pharmacological agents to treat and reduce CSB. Therefore, the databases PubMed and Google Scholar were searched for relevant studies using the following key words: *hypersexual, sexual addiction, compulsive sexual behaviour, impulsive sexual behaviour, paraphilia* AND *medication, antiandrogens, antidepressants, SSRI, naltrexone, cyproterone acetate, medroxyprogesterone acetate, pharmacological, LHRH agonist, GnRH agonists.* There was no time limit. Studies were included in case they contained original data on the treatment effectiveness of any kind of pharmacological agent in individuals with (non-paraphilic) CSB. Studies reporting only on patients with paraphilic CSB were not included because these studies have been included in the guidelines concerning the biological treatment of paraphilic disorders updated in 2020 (Thibaut et al. 2020). However, because paraphilias and CSB can be seen as closely related constructs and appear comorbid in many individuals, when formulating the current guidelines, we also reference the studies that were already included in the WFSBP (World Federation of Societies of Biological Psychiatry) guidelines on the biological treatment of paraphilic disorders, and studies concerning chemsex were also included. Altogether, there were few controlled studies, and most studies were case reports. In 2022 a placebo-controlled, double-blind, randomised controlled trial (RCT) comparing the tolerability and efficacy of paroxetine and naltrexone for treatment of compulsive sexual behaviour disorder was published, representing a milestone in the current empirical literature (Lew-Starowicz et al. 2022).

### Naltrexone (five case reports, three open studies, one RCT)

As neural differences in the processing of sexual-cue reactivity have been reported in individuals with CSB in regions previously implicated in drug-cue reactivity studies, treatments used for illicit drug- or alcohol-addiction have also been explored in patients with CSB and those with paraphilic disorders (Thibaut et al. 2020). Naltrexone is a long-acting μ-preferring opioid antagonist (i.e., it competitively blocks μ, δ, and κ opioid receptors, with 10–25-fold higher affinity for μ relative to δ and κ) used in the treatment of alcohol or opioid use disorders (Ray et al. 2019). Naltrexone may operate by inhibiting the capacity of endogenous opioids to trigger dopamine release in the nucleus accumbens in response to rewards and thus help extinguish reinforcement and addictive potential (Weerts et al. 2008). Opioid receptors are also located on GABAergic interneurons that inhibit ventral tegmental area dopaminergic neurons and dopamine signalling of the nucleus accumbens. A gradual desensitisation obtained with naltrexone could be associated with decreased pleasurable effects, potentially helping individuals with CSB reduce and regain control of their sexual behaviours (Savard 2021).

We identify five case reports and three open studies assessing the efficacy of naltrexone in reducing CSB (see Table 2).

**Table 2.** Treatment studies for naltrexone.

Reference Type of study	Patient characteristics	Previous or concurrent treatments for CSBD	Treatment conditions (dose and duration)	Outcome measures	Efficacy
**Double-blind studies**					
Lew-Starowicz et al. (2022) Poland	*N* = 73 heterosexual men (mean age 35.74 years; SD 8.09 years) with CSBD according to ICD-11; 87.7% with problematic pornography use and 100% with compulsive masturbation. No significant differences with respect to CSBD symptoms or demographic characteristics. 15 patients were suffering from sexual dysfunctions, mostly erectile dysfunction.	No previous psychiatric treatment	Group A: Paroxetine 20 mg/d Group B: Naltrexone 50 mg/d Group C: Placebo	Primary outcome: Changes in the severity of CSBD symptoms measured with SAST-R, BPS, and HBI. Additionally, craving for sexual activity and pornography vieweing were assessed via smartphone-based Ecological Momentary Assessments.	Significant decrease of self-assessed CSBD symptom severity after 8 and 20 weeks across all three groups, however, no differences between paroxetine, naltrexone and placebo. In clinical interviews paroxetine and naltrexone were found to be more effective than placebo in reducing CSBD symptoms after 8 as well as after 20 weeks. Craving for both sexual encounters and pornography viewing was reduced in the paroxetine condition but not in the other two conditions.
**Open studies**					
Ryback (2004)	*N* = 21 juvenile, legally-adjudicated sexually-offending patients (in-patients) who met any of the self-reported following criteria: (1) excessive masturbation (>3 times per day); (2) feeling unable to control arousal; (3) spending more than 30% of awake time in sexual fantasies; or (4) having sexual fantasies or behaviour that regularly intruded into and interfered with their functioning in the treatment program (13–17 years of age), high rates of co-occurring ADHD (52%), depressive disorders (24%), and substance use disorders (24%)	Almost all patients were additionally treated with other medication: 52% stimulants, 38% antidepressants, 24% risperidone	Naltrexone was started at 50 mg/d in every patient, with average dose achieved being 170 mg/day (range: 100–200 mg/day)	Outcome measures were self-reported daily sexual fantasies and frequency of masturbation. A positive result was recorded if there was more than a 30% decrease in any self-reported criterion and if this benefit lasted, at least, 4 months.	No clear benefits at doses less than 100 mg/day. At 150–200 mg/day, 15 patients (71.4%) reported benefit. Masturbation frequency decreased from two times a day to two times a week. Also decreases in durations of time fantasising about sex were noted. Those six patients who did not respond to naltrexone were changed to leuprolide (3.5 mg or 7.5 mg/month). In 5 patients, stable benefit was observed at 7.5 mg IM monthly. 13 patients had naltrexone stopped after 2 months, which resulted in reoccurrence of symptoms that began when the tapered dose reached 50 mg per day.
Raymond et al. (2010) USA	*N* = 19 patients with paraphilic and non-paraphilic CSB Mean age 44.1 years (SD = 9.4 years, range 28–62 years). Co-occurring disorders (*n*): Major depression (13), dysthymia (1), depression not otherwise specified (1), Bipolar II disorder (2), cyclothymic disorder (1), generalised anxiety disorder (5), alcohol abuse (2), cannabis abuse (1), adjustment disorder (1).	Concomitant CBT in all patients; 17 patients with concomitant antidepressant treatment	Naltrexone, dosage range 50–200 mg/d. 15 Patients concurrent use of SSRI or SNRI, two patients additionally bupropion, three patients additionally bupropion and SSRI. 15 Patients were on the same medication regime for at least 6 months at the time naltrexone was initiated. 1 patient started sertraline 1 month before naltrexone. 3 patients had stopped taking dextroamphetamine, lamotrigine, and nefazodone the day naltrexone was started.	CSB rated based on the Clinical Global Impression (CGI) score	17 patients reported that CSB symptoms improved either very much or much after naltrexone treatment was started. Mean effective dose was 104 mg/d (SD = 41 mg/d). Mean treatment duration in participants who responded to treatment was 1 year (SD = 1.0 year).
Savard et al. (2020) Sweden	*N* = 20 men (mean age 38.8; SD = 10.3) with CSBD according to ICD-11; 95% with excessive masturbation. Exclusion criteria: 1.) alcohol dependence, use of illicit drugs in the past month, ongoing opioid or benzodiazepine medication, 2.) Severe psychiatric disorder, 3.) Change of concurrent medication or dosage in the last 3 months, 4.) Sexual behaviours with high risk to offend, 5.) Ongoing psychotherapeutic treatment. Co-occurring psychiatric disorders (*n*): Anxiety disorder (5), ADHD (5),	Antidepressants (2 patients) Stimulants (1 patient)	Starting dose 25 mg for 3–5 days, then increase to 50 mg/d Naltrexone 50 mg/day for 4 weeks (25 mg/day in 1 case due to fatigue)	Hypersexual disorder: Current assessment scale (HD:CAS) Hypersexual Behaviour Inventory (HBI) Sexual compulsivity scale	Significant reduction in HD:CAS scores after 2 and 4 weeks and HBI scores after 2 weeks and after 4 weeks. Increase of scores after 4 weeks without naltrexone; however, scores were below baseline scores.
**Case studies**					
Grant and Kim (2002) USA	*N* = 1 man (58 years) Insatiable demand for multiple sexual partners and co-occurring kleptomania	Cognitive-behavioural therapy for 10 years Fluoxetine 80 mg/day for 16 weeks, both without change	Naltrexone 150 mg/day	Self-report	After 2 weeks with naltrexone at 150 mg/day, cessation of compulsive sexual behaviour reported. 3 days after treatment discontinuation, return of sexual urges reported. 4 days after restarting medication, remission of symptoms reported.
Raymond et al. (2002) USA	a.) *N* = 1 woman (42-years) frequent sexual activity with multiple partners outside her marriage; co-occurring depression b.) *N* = 1 man (62 years) extramarital affairs with work associates and prostitutes, preoccupation with masochistic fantasies	a.) 20–60 mg fluoxetine with lowering depressive symptoms b.) 40 mg/day fluoxetine	a.) Additionally 50–150 mg naltrexone/day b.) Additionally 50–100 mg naltrexone/day	Self-report	a.) With 100 mg /day naltrexone, almost complete remission of sexual urges; after 8 months, change from fluoxetine to citalopram at 60 mg/day; with increases in sexual urges, increased to 150 mg naltrexone, with subsequent decrease in sexual urges b.) With 40 mg/day fluoxetine, improvement in CSB, depressive symptoms and sexual preoccupation; due to difficulties in sexual functioning, fluoxetine switch to nefazodone. After stopping all medications, sexual preoccupation reoccurred; trials with fluoxetine, buproprion, citalopram and buspirone were ineffective. With 50 mg naltrexone, improvement in all symptoms; after 2 months, loss of efficacy; with naltrexone increase to 100 mg/day, improvement of symptoms for the next 8 months reported.
Bostwick and Bucci (2008) USA	*N* = 1 man (24 years) Preoccupation with internet pornography, multiple hours each day with sexual chats, consumption of pornography and meeting cyber-contacts for spontaneous sex.	Sertraline 100 mg/day for 1 year, group and individual psychotherapy, Sexual Addicts Anonymous, pastoral counselling	100 mg/day Sertraline + 150 mg/day naltrexone for 3 years	Self-report	After adding naltrexone, complete control over sexual impulses reported. Later, 50 mg/day achieved similar positive results.
Kraus et al. (2015) USA	*N* = 1 man (in his 30s) with problematic internet pornography viewing	Cognitive-behavioural therapy (CBT)	After 10 weeks of CBT, 50 mg/day naltrexone added given persistent/increased urges for viewing pornography	Self-report	After 2 weeks of naltrexone treatment, decreases in urges to masturbate to pornography reported.
Camacho et al. (2018) Portugal	*N* = 1 (27 years) with distressing sexual urge to have sex with transvestite men and excessive viewing of pornography (3–10 h/d)	Psychotherapy fluoxetine 20–40 mg/d, aripiprazole 10 mg/d, other antidepressants, mood stabilisers, and antipsychotics	Naltrexone 50 mg/d was added to fluoxetine 40 mg/d and aripiprazole 10 mg/d. Naltrexone was increased after several weeks to 100 mg/d.	Self-report Y-BOCS-II compulsions score	After 2 months of naltrexone treatment, significant improvement in the reduction of sexual fantasies and control of sexual impulses. No more visits of prostitutes reported. Y-BOCS-II score decreased from 14 to 0 after 2 months. After about 10 months, spontaneous stopping of naltrexone treatment and sexual compulsions returned within 2 days.

#### Case reports

Grant and Kim (2002) described a 58-year-old man who suffered from kleptomania since he was 11 years old. Approximately at the age of 50 years, the patient started to exhibit CSB. The patient reported an ‘insatiable demand for multiple sexual partners’ and multiple attempts to stop the behaviour were unsuccessful. He had previously been treated with behavioural therapy for 10 years and had taken fluoxetine (80 mg/day) for 16 weeks without any behavioural changes. Naltrexone treatment was initiated starting at a dose of 25 mg/day after fluoxetine treatment was ended. When the dose was increased to 100 mg/day, an initial reduction in his urges to steal and to engage in CSB were observable. After two weeks on 150 mg/day his stealing and CSB had ceased completely; however, he was still able to have sexual intercourse with his wife. After six weeks, the patient discontinued treatment, and within 3 days, the symptoms returned. The patient restarted treatment and after another 4 days was free of stealing and CSB again.

Raymond et al. (2002) described two patients reporting non-paraphilic CSB. The first patient was a 42-year-old woman who showed frequent sexual activities with multiple partners outside of her marriage. She felt that she had lost complete control over her sexual behaviours. The patient was diagnosed with CSBD and major depression and was treated with fluoxetine in doses up to 60 mg/day, which improved her depressive symptoms without any changes in CSB. Naltrexone treatment was started and increased up to 100 mg/day as an augmentation. After two weeks, the patient reported an almost complete remission of sexual urges. Due to sedation effects, after 8 months fluoxetine was replaced with citalopram (60 mg/day); during the switch, naltrexone was increased to 150 mg/day due to an increase in sexual urges. Then, due to sedation effects, the patient switched back to fluoxetine (10 mg/day) with naltrexone continued (150 mg/day). At 1 year, the efficacy of the combination of fluoxetine and naltrexone in treating CSB remained. The second patient was a 62-year-old man who reported frequent extramarital affairs with work associates and prostitutes. He had already been treated with fluoxetine 40 mg/day and showed some improvement in CSB and depressive symptoms. Because of sexual dysfunction he stopped fluoxetine treatment; however, intense sexual preoccupation returned. The use of fluoxetine, bupropion, citalopram and busipirone was ineffective. Naltrexone treatment at 50 mg/day was initiated to augment citalopram (40 mg/day), and the patient reported that after 1 month of treatment, the obsessive thoughts about sexual acting out were gone. After a short reoccurrence of intrusive sexual thoughts, the dose was increased to 100 mg/day and the symptoms vanished again. No liver dysfunction was reported.

A male 24-year-old patient presented to a clinic with an intense preoccupation with internet pornography (Bostwick and Bucci 2008). The patient described that he spent many hours each day chatting online, engaging in extended masturbation sessions, and meeting cyber-contacts in person for spontaneous sex. The patient reported he had previously been treated with antidepressants, group and individual psychotherapy, and had attended Sexual Addicts Anonymous and pastoral counselling, all without effect. Due to depressive symptoms the patient was treated with sertraline 100 mg/day, however, without an effect on CSB. Naltrexone treatment was started as an augmentation at a dose of 50 mg/day, and by 1 week later, the patient reported a measurable difference in sexual urges. However, the patient did not achieve full control over his sexual urges until the dose was increased to 150 mg/day. The symptoms disappeared when naltrexone was added to sertraline and reappeared when naltrexone was decreased to less than 50 mg/day.

Kraus et al. (2015) described the case of a male veteran in his 30 s who suffered from compulsive masturbation to pornography for the previous 10 years. The patient started with weekly sessions of CBT, which led to a 70% decrease in pornography consumption; however, sexual urges to masturbate to pornography continued. After naltrexone (50 mg/day) was initiated, the patient reported decreased urges to masturbate to pornography with persisent/further decreases in frequency of pornography viewing.

Camacho et al. (2018) described a 27-year-old man who reported having spent a significant amount of time and money fantasising about and hiring prostitution services. The patient had a particular fixation with ‘transvestite men’. The patient further indicated that he felt unable to control these sexual fantasies and behaviours. Besides having sex with transvestite men about once every 2 months, the patient also reported excessive pornography consumption lasting between 3 and 10 h a day. He was being treated with fluoxetine 40 mg/day and aripiprazole 10 mg/day and psychotherapy, without positive effect. Two months after naltrexone (50 mg/day) was added to fluoxetine and aripiprazole, the patient reported significant improvements in the reduction of sexual fantasies and control of sexual impulses. Even after fluoxetine and aripiprazole were stopped, benefits remained. After a couple of months, naltrexone was increased to 100 mg/day. After 10 months of treatment, the patient spontaneously stopped naltrexone treatment, and within 2 days, sexual fantasies and urges increased again, so he resumed the treatment (Camacho et al. 2018).

#### Open uncontrolled studies

Ryback (2004) studied the efficacy of naltrexone in 21 adolescents between 13 and 17 years of age who had been convicted of sexual offences and showed symptoms of CSB (note that sexual fantasies were often of paraphilic content). Naltrexone treatment was started with 50 mg/day in every patient and the average maintenance naltrexone dose was 170 mg/day (range: 100–200 mg/day). No benefit was observed at naltrexone dosages of less than 100 mg/day. At dosages between 150 mg/day and 200 mg/day, 15 patients reported a benefit. Dosages above 200 mg/day did not lead to additional benefit.

Raymond et al. (2010) retrospectively rated the medical charts of 19 patients (mean age 44.1 years, SD = 9.4 years) with paraphilic (*n* = 8) and non-paraphilic (*n* = 11) CSB. In total, 17 patients reported significant reductions in CSB symptoms after naltrexone treatment was initiated. The mean effective naltrexone dose for these 17 patients was 104 mg/day (SD = 41 mg/day).

In the largest uncontrolled study using naltrexone to treat CSB, Savard et al. (2020) treated a sample of 20 men with non-paraphilic CSBD from Sweden. Inclusion criteria were a CSBD diagnosis according to the ICD-11 diagnostic criteria. Naltrexone 50 mg/day was used in all cases except for 1 case where naltrexone was reduced to 25 mg/day due to fatigue. Treatment duration lasted four weeks in all patients. A significant reduction of CSB assessed with the hypersexual disorder: current assessment scale (HD: CAS), the HBI-19 and the SCS was found after two and four weeks of treatment, with a large effect size. Altogether 19 patients reported at least one adverse event, most frequently fatigue (55%), nausea (30%), vertigo (30%), abdominal pain (30%), and apathy (15%); however, only three patients experienced adverse effects over the four-week treatment period.

#### Double-blind placebo-controlled study

Lew-Starowicz and colleagues just recently published the results of the first RCT comparing the tolerability and efficacy of naltrexone and paroxetine in comparison to placebo (Lew-Starowicz et al. 2022). They included 73 heterosexual men (mean age = 35.7 years; SD = 8.1 years) who were randomly assigned to be treated with either naltrexone (50 mg/d), or paroxetine (20 mg/d), or placebo over a period of 20 weeks. All participants were diagnosed with CSBD according to ICD-11 diagnostic criteria. Most participants reported about problematic pornography use and compulsive masturbation. Self-reported severity of CSBD symptoms significantly decreased over time, however, there was no difference between the treatment conditions. Participants from both the paroxetine and naltrexone conditions reported a reduced frequency of sexual binges at week 20 and a decrease in the frequency of CSBD symptoms compared to the placebo condition. Furthermore, only in the paroxetine condition a significant reduction of craving symptoms could be observed at the end of the treatment period.

Altogether, five patients dropped out during the trial due to side effects. Most frequently occurring side effects were sedation (paroxetine 29.2%; naltrexone 37.5%) and orgasmic dysfunction (20.8%). All other side effects were similar to reports on safety and tolerability profiles of paroxetine and naltrexone in their registered indications.

#### Naltrexone adverse effects and contra-indications

Adverse effects with naltrexone include specific concerns in the following domains. Digestive: nausea (10%), vomiting (3%), anorexia, diarrhoea, constipation, abdominal pain. Psychiatric: asthenia, irritability, depression (15%), suicidal thoughts (1%), anxiety, insomnia. Others: headache (7%), akathisia, dizziness (3%), thirst, sweating, chills, sexual dysfunction, delayed ejaculation, arthralgia, myalgia, rash, tachycardia, palpitations, weight loss, chest pain, electrocardiogram changes. If >50 mg/day: increase in transaminases.

The most common adverse effects of naltrexone observed in the treatment of alcohol-use disorder are gastrointestinal (e.g., nausea reported by approximately 10%), sedative effects, and headache (approximately 7%), and they usually subsided within 1 week (Rösner et al. 2010). A maximum studied duration of treatment is 3 months in the maintenance of abstinence in alcohol-use disorders, although longer durations occur in clinical practice.

Contra-indications: acute hepatitis or severe hepatocellular insufficiency; concomitant use of opioids; pregnancy, lactation; suicidal risk; severe kidney failure; hypersensitivity to naltrexone or one of its components; individuals under 18 or over 65 (French Health Authority 2015).

### Selective serotonin reuptake inhibitors (two case reports, four open studies and two RCTs)

In the past 30 years, numerous case reports and open studies have described the efficacy of SSRIs in the treatment of some paraphilic disorders, as well as non-paraphilic CSB, although no formal indications exist for SSRIs for these conditions (Kafka and Prentky 1992; Garcia and Thibaut 2010; Thibaut et al. 2020). One proposed mechanism of action relates the anti-obsessional effects of SSRIs to the hypothesis that CSB and some paraphilias may be related to OCD or impulse-control disorders. It has been proposed that the increased binding of the neurotransmitter serotonin to 5-HT-2 receptors in the brain and spinal cord achieved by SSRIs may lead to a general decrease in sexual drive, reduced erectile functioning and delayed ejaculation (Waldinger et al. 1998; Pfaus 2009). When used in the treatment of mood disorders, sexual dysfunctions related to the use of SSRIs have been reported, with prevalence estimates between 20% and 70% of patients, and such dysfunction may be more persistent over longer courses of treatment (Serretti and Chiesa 2009) (Table 3).

**Table 3.** Treatment studies for SSRIs.

Reference Type of study	Patient characteristics	Previous or concurrent treatments for CSBD	Treatment conditions (dose and duration)	Outcome measures	Efficacy
**Double-blind studies**					
Wainberg et al. (2006) USA	*N* = 28 homosexual men (mean age 36.8 years) with CSBD	23% psychotherapy, 3.8% medication, 19.2% self-help groups	13 Participants citalopram (20–60 mg/d) vs. 15 participants placebo for 12 weeks. Mean daily citalopram dose at week 4 was 25.5 mg and 43.36 mg at week 12.	Clinical Global Impressions scale-Compulsive Sexual Behaviour (CGI-CBS), Compulsive Sexual Behaviour Inventory (CSBI), Yale-Brown Obsessive Compulsive Scale-Compulsive Sexual Behaviour (YBOCS-CSB)	Decreases were observed in CSB symptoms in both groups after 12 weeks. However, stronger decreases in desire for sex, frequency of masturbation, and hours of pornography use per week were observed in the citalopram group. Although there was a significant decrease in CSB symptoms measured with the YBOCS-CSB, the Compulsive Sexual Behaviour Inventory or the CGI-CSB in the whole group, no differences were observable between the two groups. Furthermore, although there was a significant decrease in the number of partners per month and in the frequency of oral and anal sex per month across the whole study population, again no differences occurred between the two groups. The same pattern was found concerning frequency of risky sexual contacts per month.
Lew-Starowicz et al. (2022) Poland	*N* = 73 heterosexual men (mean age 35.74 years; SD 8.09 years) with CSBD according to ICD-11; 87.7% with problematic pornography use and 100% with compulsive masturbation. No significant differences with respect to CSBD symptoms or demographic characteristics. 15 patients were suffering from sexual dysfunctions, mostly erectile dysfunction.	No previous psychiatric treatment	Group A: Paroxetine 20 mg/d Group B: Naltrexone 50 mg/d Group C: Placebo	Primary outcome: Changes in the severity of CSBD symptoms measured with SAST-R, BPS, and HBI. Additionally, cravings for sexual activity and pornography vieweing were assessed via smartphone-based Ecological Momentary Assessments.	Significant decrease of self-assessed CSBD symptom severity after eight and 20 weeks across all three groups, however, no differences between paroxetine, naltrexone and placebo. In clinical interviews paroxetine and naltrexone were found to be more effective than placebo in reducing CSBD symptoms after eight as well as after 20 weeks. Craving for sexual encounters and pornography viewing was reduced in the paroxetine condition but not in the other two conditions.
**Open studies**					
Kafka (1991) USA	*N* = 10 men with nonparaphilic sexual addictions (5 men with additional paraphilic disorders) (age range 27–50 years)	Supportive psychotherapy	6 Patients fluoxetine 20–60 mg/day, 1 patient imipramine 225 mg/day, 1 patient lithium 1500 mg/day, 1 patient imipramine 125 mg/day + lithium 600 mg/day, 1 patient fluoxetine 60 mg/day + trazodone 150 mg/day for 12 weeks	Sexual Outlet Inventory	All but one patient (fluoxetine monotherapy) experienced substantial improvement in symptomatology.
Kafka and Prentky (1992) USA	*N* = 10 men with paraphilic disorder and non-paraphilic sexual addictions vs. *N* = 10 men with non-paraphilic sexual addictions only 11 men (6 paraphilic and 5 non-paraphilic) met criteria for current major depression using DSM-III-R criteria	10 Men concurrent psychotherapy	Fluoxetine starting at 20 mg/day to a maximum of 60 mg/day. Mean dose at 12 weeks was 39.37 mg/day (SD = 14.81)	Sexual Outlet Inventory	4 men dropped out during the study (1 paraphilic and 3 non-paraphilic) Significant reductions were observed in total sexual outlet, masturbation frequency, frequency of sexual activities, intensity of sexual desire after 12 weeks of treatment. Greater decreases were observed in the paraphilic group in total sexual outlet; however, higher baseline total sexual outlet was observed in the paraphilic group. Overall average reduction of total sexual outlet was 65.2%
Stein et al. (1992) USA	*N* = 13 men (5 with paraphilic disorder, 5 with non-paraphilic sexual addictions, 3 with sexual thoughts or rituals that met DSM-III-R criteria for obsessive compulsive disorder (OCD); ages between 18–58 years)	Not reported	Fluoxetine up to 80 mg/d; clomipramine up to 400 mg/d Fluvoxamine up to 300 mg/d; Fenfluramine up to 40 mg/d	Clinical Global Impression change score	No change was reported in paraphilic fantasies or behaviours or improvement in non-sexual OCD symptoms; in 2 of 5 patients, decreases reported in non-paraphilic sexual addiction; in 2 of 5 improvement noted in sexual obsessions or compulsions
Kafka (1994) USA	*N* = 12 men with paraphilia-related disorders (mean age 39.6 years; SD = 7.5 years)	Not reported	Sertraline mean dose 99.0 mg, SD = 62.8 mg/day (range 25–250 mg/day) for 4–64 weeks	Sexual Outlet Inventory	Statistically significant reduction in unconventional total sexual outlet and average time spent per day without adversely affecting conventional total sexual outlet.
**Case studies**					
Elmore (2000) USA	a.) *N* = 1 man (24 years) with excessive use of telephone sex, excessive masturbation and co-occurring depression b.) *N* = 1 woman (30 years) with excessive masturbation and compulsive sex with multiple partners, co-occurring mixed personality disorder and PTSD	Not reported	In a.) Paroxetine 20 mg/day for 4 months In b.) Sertraline 200 mg/day for 20 months, partly with trazodone augmentation 150 mg/day for 6 months, amitriptyline augmentation 50 mg/day for 1 year	Self-reported intensity and frequency of CSBs	In a.) Decreases in CSBs after 2 weeks In b.) Decreases in sexual desire, arousal, and sexual activity.
Gola and Potenza (2016) Poland	*N* = 3 men (24, 32, and 35 years) Problematic pornography use and compulsive masturbation	Cognitive-behavioural therapy (on-going)	Paroxetine 20 mg/day Treatment duration between 14.5 and 20.5 weeks Follow-up 3-month	Self-reported time spent with pornography consumption (daily)	Decreased libido and delayed ejaculation during first 2–4 weeks. Within 10 weeks, normalisation of libido and sexual functioning. After 12 weeks, occurrence of new sexual behaviours (e.g., engaging in paid sexual relationships, extra-marital affair); however, no increase in pornography consumption. Still no increase in pornography consumption after 3-month follow-up

#### Case reports

Elmore described a 24-year-old man with depression and CSB symptoms (Elmore 2000). The patient masturbated two to six times a day and made excessive use of sex hotlines. After initiation of paroxetine at a daily dose of 20 mg, the patient observed a significant decrease in his CSB symptoms. No adverse effects were observed.

In another case series of three heterosexual men from Poland, Gola and Potenza (2016) found a significant treatment effect of paroxetine at a dose of 20 mg/day on PPU. The three patients were 24, 32, and 35 years of age respectively, viewed between 6.5 and 12 h pornography a week, and masturbated between 8 and 18 times a week. None of the patients took any medication and all received CBT in addition to paroxetine. During the initial two to four weeks, all patients reported about decreased sex drive and delayed ejaculation; however, these adverse effects diminished within the 10-week study period. Although the frequency of pornography consumption decreased, this effect was not statistically significant. Anxiety also decreased. After 12 weeks of treatment, all three patients started engaging in new sexual behaviours (paid sexual relationships and an extra-marital affair). While these new sexual behaviours were experienced as more or less ego-syntonic and there was no increase in the amount of pornography consumption or in the frequency of masturbation, the reports raise concerns regarding the emergence of problematic sexual behaviours when targeting PPU with paroxetine.

#### Open uncontrolled studies

In a series of open studies assessing the effectiveness of SSRIs in the treatment of paraphilic and non-paraphilic CSB, Kafka (1991) reported in a first study about 10 men of whom six were treated with fluoxetine (dose between 10 mg/day to 60 mg/day), one was treated with imipramine (125 mg/day), one with impramine (225 mg/day) and lithium (600 mg/day), one with lithium (1500 mg/day), and one with fluoxetine (60 mg/day) together with trazodone (150 mg/day). In all but one patient (fluoxetine monotherapy), a considerable decrease of the total sexual outlets was reported. Another 20 men (*n* = 10 with paraphilic disorders, *n* = 10 with CSBD) were treated with fluoxetine monotherapy at a mean dose of 39.37 mg/day over a period of 12 weeks (Kafka and Prentky 1992). In both groups, the mean total sexual outlets significantly decreased over the course of the 12 weeks. This decrease was greater in the group of the paraphilic patients; however, the paraphilic patients also had a significantly higher total sexual outlets at baseline. In the last study of his series, Kafka (1994) described a group of 26 men (*n* = 14 with paraphilic disorders, *n* = 12 with CSBD), of whom 24 were treated with sertraline for at least four weeks. The mean sertraline dose was 99.0 mg/day (SD = 61.8 mg/day). Four men received antidepressant augmentation with methylphenidate, trazodone, or lithium. A significant reduction in unconventional total sexual outlets was observed during sertraline treatment, and such an effect was not observed for conventional total sexual outlets. Nine men with an unsatisfactory clinical response to sertraline were subsequently treated with fluoxetine at a mean dose of 51.1 mg/day (SD = 19.6 mg/day). Of these men, one non-paraphilic CSBD patient improved very much, one improved much, two improved minimally, and one did not improve at all.

Stein et al. (1992) retrospectively reviewed the charts of 13 patients who were treated with a SSRI because of a paraphilic disorder, a CSBD or sexual obsessions. The following SSRIs were used within the clinical trial: fluoxetine (up to 80 mg/day), clomipramine (up to 400 mg/day), and fluvoxamine (up to 300 mg/day). Only in two of the paraphilic patients did SSRI treatment led to a reduction in CSB, mainly decreased masturbation. A similar proportion was found for the CSBD patients with three non-responders within this group. In two of the three patients with sexual obsessions and compulsions, SSRI treatment led to a reduction in these symptoms.

#### Double-blind placebo-controlled studies

In the first double-blind, placebo-controlled study of SSRIs for CSBs published to date, the authors compared a group of 13 homosexual or bisexual men being treated with a flexible dose of 20 mg/day to 60 mg/day of citalopram with a group of 15 homosexual or bisexual men being treated with placebo over a period of 12 weeks (Wainberg et al. 2006). After 12 weeks of treatment, there was a significantly stronger decrease in the desire to have sex, in masturbation frequency per week, and in hours spent viewing pornography per week in the citalopram group compared to the placebo group. Men in the citalopram group reported delayed ejaculation significantly more often than those from the placebo group.

For the second RCT, published just recently, please refer to section ‘Double-blind placebo-controlled study’.

#### SSRI adverse effects and contra-indications

Common adverse effects of SSRIs include: feelings of agitation, indigestion, diarrhoea or constipation, dizziness, blurred vision, dry mouth, excessive sweating, sleeping problems, headaches, loss of libido, erectile dysfunction, delayed ejaculation, QT prolongation (Laux 2020). Especially at the beginning of SSRI treatment, suicidal thoughts can increase. While most of these side effects improve over time, some can persist, especially sexual problems (Bala et al. 2018). In older adults, SSRI treatment can lead to severe hyponatraemia.

Caution is warranted when combining SSRIs with other medications that may increase serotonin levels due to the risk of serotonin syndrome. Serotonin syndrome can be a potentially life-threatening condition and can include symptoms such as confusion, agitation, muscle twitching, shivering, diarrhoea, fever, seizures, arrhythmia, and loss of consciousness. Contra-indications for SSRIs include acute manic states, liver and kidney diseases, and long-QT syndrome (Laux 2020).

### Other medications – clomipramine (one case report)

#### Case reports

In their case report, Rubey et al. (1993) described their treatment experience using clomipramine in a 25-year and a 19-year-old patient with severe sexual preoccupations and sexual behaviours (see Table 4). The first patient reported a 10-year history of daily masturbation, visits to prostitutes several times per week and an intense preoccupation with pornography. There were no comorbid psychiatric disorders. Two weeks after initiation of clomipramine at 125 mg/day, the patient reported decreases in time spent thinking about sex and masturbation frequency. However, the patient also suffered from considerable delayed ejaculation up to anorgasmia and subsequently stopped taking the medication. After termination of treatment, the patient experienced symptoms of CSB again, and treatment was restarted leading to a rapid decrease in CSB. The second patient had borderline mental retardation (IQ 65) and reported a constant need for sex. While inpatient, the individual exhibited repeated inappropriate sexual behaviours towards female staff and was treated with clomipramine 150 mg/day. A decrease in sexual thoughts and behaviours was noted after 18 days of treatment. Adverse effects were not reported.

**Table 4.** Treatment studies with other drugs.

Reference Type of study	Patient characteristics	Previous or concurrent treatments for CSBD	Treatment conditions (dose and duration)	Outcome measures	Efficacy
**Open studies**					
Coleman et al. (2000) USA	*N* = 14 men (age range 26–67 years) with CSBD diagnoses; 5 patients with co-occurring paraphilias	2 or more SSRIs	11 Men treated with nefazodone long-term treatment (13.4 months on average) with mean dose of 200 mg/d (range 50–400 mg/d)	Self-report	In six patients, good control over recurrent, intrusive sexual thoughts. In five patients, remission of CSBD symptoms.
Kafka and Hennen (2000) USA	*N* = 26 men with paraphilias (*n* = 14) and non-paraphilic hypersexuality (*n* = 12) 17 men with an additional retrospective diagnosis of ADHD and symptoms of ADHD persisting into adulthood Mean age 37.7 years	T1: Before SSRI treatment; T2: after SSRI treatment but immediately prior to the prescription of a psychostimulant; T3 follow-up of combined SSRI and psychostimulant treatment. At T1: 19 men treated with fluoxetine at a mean dose of 49 mg/day, 3 men treated with sertraline at a mean dose of 110 mg/day, 2 men treated with paroxetine at a mean dose of 35 mg/day, 2 men treated with fluvoxamine at a mean dose of 100 mg/day; SSRI treatment on average 8.8 months.	At T2: 25 men treated with methylphenidate sustained release with mean dose of 40 mg/day (range 20–100 mg/day) and 1 participant dextroamphetamine for at least 8 weeks	Sexual Outlet Inventory	21 Men completed the trial. At T2: Significant reduction in total sexual outlet and median time per day spent in sexual behaviours. At T3: Significant reduction in total sexual outlet and time spent with sexual behaviours per day. No difference between paraphilic and non-paraphilic hypersexual patients on outcome measures. After 8 weeks both the total sexual outlet and the median time per day spent with sexual behaviours were substantially decreased. Total sexual outlet decreased more than 63% from 9 to 3.
**Case studies**					
Rubey et al. (1993) USA	*N* = 2 men a.) 25 years; daily masturbation, prostitutes several times a week, preoccupation with pornographic material b.) 19 years; borderline mentally retarded, intrusive sexual thoughts	Not reported	a.) Clomipramine 125 mg/day b.) clomipramine 150 mg/day	Self-report	a.) After 1 week, decline in sexual ruminations and almost no urge to visit prostitutes or pornographic bookstores, decline in masturbation frequency b.) Decrease in sexual thoughts
Fong et al. (2005) USA	*N* = 1 man (32 years) Obsessive use of strip clubs and massage parlours	Cognitive-behavioural therapy and fluoxetine 80 mg/d for 6 months, no change in sexual symptoms, naltrexone 25 mg was added to fluoxetine; however, this was stopped after 10 days due to nausea.	Topiramate 200 mg/d monotherapy	Self-report	After 6 weeks, stopped engaging in sexual behaviours.
Khazaal and Zullino (2006) Switzerland	*N* = 1 man (33 years of age) Excessive sex with prostitutes (spending nearly one-third of monthly income) No history of other psychiatric disorder Co-occurring obesity	Simultaneously three sessions of cognitive-behavioural therapy for obesity	50 mg/day topiramate monotherapy for 4 months	Self-report	Reduction in sex with prostitutes; reductions in urges, cravings and attractions of environmental cues after two weeks of treatment. 2 weeks after discontinuation of treatment, relapse in compulsive visits with prostitutes
Gulsun et al. (2007) Turkey	*N* = 1 woman (21 years of age) Compulsive telephone sex accompanied by masturbation (15–16 times a day) No history of any other medical condition	None	First 3 months: clomipramine 150 mg/day; after 3 months, additional treatment with valproic acid 1000 mg/day	Self-reported masturbation frequency	2 weeks after treatment started with valproic acid. decrease in masturbation frequency was noted. One month later, no more masturbation reported.
Blum and Grant (2022) USA	*N* = 2? or 8 men (age range 21–56 years) with CSBD diagnosis; 4 patients with co-occuring anxiety disorders, 4 patients with co-occuring depression, and 2 patients with co-occuring substance use disorder	Additional treatment: 4 patients had an SSRI, 1 patient had naltrexone, 1 patient had phenelzine, and 1 patient had bupropion.	All patients started with 600 mg NAC twice a day, increase of dose to 1200 mg NAC twice a day after 1 week. Four patients stayed on 2400 mg daily and four patients increased the dose to 3600 mg daily. Treatment duration: 2–6 months	CSB-Yale-Brown Obsessive Compulsive Scale (YBOCS)	CSB-YBOCS scores were assessed before NAC treatment was started and 4–6 weeks into treatment. Five patients reported an improvement of more than 35% on the YBOCS. Three patients did not show an improvement.

#### Clomipramine adverse effects and contra-indications

Clomipramine is a tricyclic antidepressant. Common adverse effects include drowsiness, dry mouth, nausea, diarrhoea, constipation, nervousness, decreased sexual drive and functioning, decreased memory or concentration, and weight gain. More serious but rarely occurring side effects are seizures, tachyarrhythmia, poor bladder control, hallucinations, muscle stiffness, sore throat, and fever (Corponi et al. 2020).

Strong/absolute contra-indications include urinary retention, angle-closure glaucoma, prostate hyperplasia, ileus, delirium, myocardial infarction, and long-QT syndrome. Relative contra-indications are severe liver or kidney diseases and severe cardiac diseases (Corponi et al. 2020).

### Other medications – topiramate (two case reports)

#### Case reports

Topiramate is an anticonvulsant that has multiple sites and modes of action, including the modulation of voltage-dependent sodium and calcium ion channels, potentiation of GABA neurotransmission, and blockade of kainate/alpha-amino-3-hydroxy-5-methyl-4- isoxazolepropionic acid (AMPA) glutamate receptors. It has been shown to have promise in treating alcohol-use, binge-eating disorders, and kleptomania. It may be an ‘anti-impulsivity’ medication. The stimulation of AMPA receptors may reinstate drug-seeking and, conversely, the antagonism of AMPA receptors may block reinstatement.

Fong et al. (2005) described a 32-year-old patient who reported excessive use of strip clubs and massage parlours, spending about $2000 per week on these activities (see Table 4). He did not watch pornography. Furthermore, the patient described an increased heart rate, nervousness, dry mouth, and nausea before engaging in sexual behaviours, and these somatic symptoms diminished after the sexual behaviours. There were no comorbid psychiatric disorders. Only a small decrease in sexual activities was observed after 12 sessions of CBT. Fluoxetine treatment at a dose of 80 mg/day over a period of 6 months had no significant effect either. Naltrexone 25 mg/day was added but was discontinued due to adverse effects. Finally, topiramate treatment alone was initiated and increased to a dose of 200 mg/day over a one-month period. After six weeks of topiramate treatment at a dose of 200 mg/day, the patient reported a complete cessation of somatic symptoms prior to engaging in sexual behaviours and was able to completely stop his unwanted sexual activities. After 6 months of treatment, the patient reported increased urination, bladder fullness and dizziness with no somatic correlates. He stopped the medication and three weeks later noticed a return of sexual urges. Thus, topiramate was started again leading to a complete cessation of CSB symptoms.

Another case report involved a 33-year-old man without any history of psychiatric morbidity who was treated with topiramate 50 mg/day initially because of excessive food intake (see Table 4) (Khazaal and Zullino 2006). After 2 months of treatment, the patient reported that in addition to having better control of his eating behaviour, he could better control his excessive sexual activities as well, which he did not report about at first due to feelings of shame and guilt. Beforehand, the patient had repeatedly visited massage clubs and prostitutes, spending nearly one-third of his monthly income on these. With topiramate, his intense urges and craving to visit prostitutes diminished. After 4 months of treatment, he discontinued topiramate treatment; however, compulsive eating and compulsive sexual activities returned. Restarting topiramate led to a significant decrease in compulsive behaviours again.

#### Topiramate adverse effects and contraindications

Topiramate is an anticonvulsant. Common adverse effects include weight loss and anorexia, paresthesias, fatigue, dizziness, taste disorders, nausea, abdominal pain, diarrhoea, cognitive and amnestic disorders, tremor, balance disorders, arthralgias, glaucoma, nystagmus, metabolic acidosis, depression or psychotic symptoms, aggressiveness, increased risk of suicide, leucopoenia, and thrombocytopenia. Strong/absolute contraindications are severe liver and kidney diseases, nephrolithiasis, and dehydration (Dinkelacker et al. 2020).

### Other medications – nefazodone (one open study)

#### Open uncontrolled studies

Nefazodone is a phenylpiperazine antidepressant with a mechanism of action that is distinct from currently available drugs. It potently and selectively blocks postsynaptic serotonin 5-HT-2_A_ receptors and moderately inhibits serotonin and noradrenaline reuptake. Nefazodone is not associated with the sexual side effects of SSRIs.

Between 1995 and 1997, 14 men (aged 26–67 years, inclusively) were treated with nefazodone for non-paraphilic CSB (see Table 4) (Coleman et al. 2000). All participants were additionally treated psychotherapeutically. All patients were previously treated with a SSRI; however, SSRI treatment was terminated because of ineffectiveness or significant adverse effects. Nine patients were diagnosed with a co-occurring mood disorder and three with a co-occurring anxiety disorder. Eleven patients were treated with nefazodone on average for 13.4 months, and three patients discontinued treatment, mainly because of adverse effects (e.g., headache, bloating). The mean dose was 200 mg/day (range: 50–400 mg/day). In six patients, nefazodone treatment led to good control of recurrent, intrusive sexual thoughts, and in the remaining five patients complete remission of symptoms was reported. Only one patient reported initial dizziness that vanished after a few weeks.

#### Nefazodone adverse effects and contraindications

Adverse effects may include headache, difficulty with concentration, dry mouth, flushing, pain or burning in hands or feet, constipation, rash, itching, slow heartbeat, memory problems, blurred vision, confusion, seizures, and painful erection of the penis (Corponi et al. 2020). Contraindications include dehydration, acute manic symptoms, heart attack, angina and acute stroke.

### Other medications – psychostimulants (one open study)

#### Open uncontrolled studies

Kafka and Hennen (2000) described 26 patients either with a paraphilic disorder (*n* = 14) or a non-paraphilic CSBD (*n* = 12) (see Table 4). Of these, 21 were diagnosed with a co-occurring lifetime depressive disorder and 17 with co-occurring ADHD. SSRI treatment led to significant decrease in CSBD symptoms after on average 8.8 months (SD = 11.1 months) of treatment. After psychostimulant augmentation, another significant decrease in CSBD symptoms was reported. On average, participants were treated with an SSRI in combination with a psychostimulant for 9.6 months (SD = 8.2 months). The mean dose of methylphenidate was 40 mg/day (range 20–100 mg/day). No differences were observed either between patients with a paraphilic disorder or a CSBD or in patients with or without co-occurring ADHD. Duration of treatment also did not influence results.

#### Psychostimulants adverse effects and contraindications

Common adverse effects include headache, dizziness, sleeping disorders, nervouseness, loss of appetite, tachycardia, dry mouth, weight loss, and increased blood pressure. In rare cases, psychotic symptoms may occur. Contraindications include cardiovascular diseases (Rösler et al. 2020).

### Other medication – N-acetylcysteine (one case series)

#### Case series

Blum and Grant (2022) published a retrospective chart review of eight male patients with CSBD who were treated either with N-acetylcysteine (NAC) monotherapy or with NAC in combination with other medications (see Table 4). While three patients showed online sexual activities only, two patients showed offline sexual activities and two patients both. All patients received NAC for at least 2 months and a maximum of 6 months. Patients were started with NAC 600 mg twice daily and the dose was increased to 1200 mg twice daily after 1 week. In four patients the dose was increased after another week to 1800 mg twice a day. One patient was additionally treated with phenelzine (90md/d), three patients received an additional SSRI (either fluoxetine 20 mg/d or escitalopram 10 mg/d), one patient additionally received naltrexone and escitalopram (20 mg/d) and one patient additionally received bupropion (450 mg/d).

On average, an improvement of 40.9% in the CSB-Y-BOCS was observed across all eight patients between baseline and 4–6 weeks into treatment. Three patients did not show any improvement on the CSB-Y-BOCS. The two patients receiving NAC monotherapy showed a strong improvement in CSB symptoms (more than 35% improvement of the YBOCS). None of the eigth patients reported about severe side effects. Four patients reported about mild nausea and one patient about mild headaches.

#### N-acetylcysteine adverse effects and contraindications

Adverse effects rarely occur, however, these can include nausea, vomiting, headache, tinnitus, reflux, and allergic reactions. NAC should not be taken at the same time as certain antibiotics, for example penicilline and cephalosporines. Contraindications include an increased risk for gastrointestinal haemorrhage, asthma or a hyperactive bronchial system, and high blood pressure (Tenório et al. 2021).

### Cyproterone acetate (CPA) (one case report, one open study in patients with CSB and comorbid paraphilic disorders)

Some case reports of hormonal treatments support the use of medroxyprogesterone acetate (MPA) for the treatment of CSB and/or paraphilic behaviours in older patients with dementia (Cooper 1987; Cross et al. 2013). All patients had received other psychotropic medications before MPA treatment. Due to adverse effects, these medications are not used in Europe and are not recommended.

CPA is a synthetic steroid, similar to progesterone, which acts both as a progestogen and an antiandrogen. Direct CPA binding to androgen receptors (including those in the brain) blocks intracellular testosterone uptake and metabolism. Indeed, CPA is a competitive inhibitor of testosterone and dihydrotestosterone at androgen receptor sites. In addition, it has a robust progestational action, which causes an inhibition of GnRH secretion and a decrease in both gonaotropin releasing hormone (GnRH) and luteinizing hormone (LH) release (Rabe et al. 1996).

#### Case studies

The efficacy of CPA on CSB and/or masturbation in public was reported in several cases of adolescent males with mental retardation and other individuals (see Table 5) (Davies 1974). The definition of CSB chosen by the author is outdated and included cases of sexually offending individuals without apparent CSB, men with chromosome abnormalities or older adult men with sexual misconduct. Additionally, four patients reported intense, recurrent sexual fantasies that were clinically distressing. After initiation of CPA treatment at doses between 50 mg/day and 100 mg/day, these patients reported a markable decrease in masturbation frequency. Another ten patients also described CSB; however, treatment in these men was also initiated in order to change homosexual orientation. After start of CPA treatment, there was a significant decrease in masturbation frequency. Sexual orientation unsurprisingly did not change.

**Table 5.** Treatment studies for hormonal agents.

Reference Type of study	Patient characteristics	Previous treatments	Treatment conditions	Outcome measures	Efficacy
**Open studies**					
Winder et al. (2014,2018) UK	*N* = 127 men convicted for sexual offences currently imprisoned with hyper-arousal, intrusive sexual fantasies or urges, or dangerous paraphilias	83% in sex offender treatment program	Cyproterone acetate (CPA) at 50–100 mg/d (16 patients) Fluoxetine or paroxetine 20–60 mg/day (75 patients) SSRI + CPA (7 patients) GnRH-agonists (two patients) No medication (20 patients)	Sexual Compulsivity Scale (SCS); self-reported measures of sexual thoughts, feelings, and behaviours	Only participants that took medication for 6 months were included (*n* = 33): Significant reductions in SCS scores noted after 3 months and after 6 months. Significant reductions in masturbation frequency, time spent thinking about sex, and strength of sexual urges reported. Ability to distract from sexual thoughts already after 1 month (stronger decrease during SSRI than during CPA treatment).
**Case studies**					
Davies (1974)	a.) *N* = 2 men with distressing vivid sexual fantasies 1 Man (22 years) with additional sadistic fantasies 1 Man (33 years) with excessive masturbation b.) *N* = 10 homosexual men with CSB	Not reported	Cyproterone acetate 50–200 mg/day; Treatment duration not reported	Masturbation frequency	In a.) reductions in masturbation frequency and disappearance of sadistic fantasies. In b.) reduction in masturbation frequency.

#### Open uncontrolled studies

Winder and colleagues reported on a sample of 127 imprisoned men who had been convicted for a sexual offence and exhibited CSB (Winder et al. 2014,2018). Of these patients, 75 received a SSRI (mainly fluoxetine at a dose between 40 and 60 mg/day), 16 patients received CPA (between 50 and 100 mg/day), seven patients were treated with a combination of SSRIs and CPA, and two patients were treated with a GnRH agonist. Only 33 patients continued to take the prescribed medication for at least 6 months. Across all included patients, there was a significant decrease in CSB after 3 months of treatment. SSRIs led to a faster decrease compared to CPA with respect to decreasing sexual thoughts.

#### CPA adverse effects and contraindications

CPA treatment can be accompanied by diverse mild and severe side effects which may relate to reduced testosterone concentrations. Observed adverse effects may include weight gain, hot flushes, pain at the site of injection, lethargy and depression, gynaecomastia, thromboembolic events and liver and kidney dysfunction (Assumpcao et al. 2014). Most adverse effects are reversable once treatment has been terminated. Recently, it has been reported that CPA treatment is associated with a risk of meningioma. Thus, CPA should not be used in patients with severe liver and kidney diseases or in patients with meningiomas.

### GnRH analogues

Via stimulation of GnRH receptors in the pituitary gland, GnRH agonists (synonym: LHRH agonists) can promote marked decreases in the sensitivity and in number of the corresponding receptors. As a result, the secretion of LH may decrease significantly. Due to the diminshed/lack of LH secretion from the pituitary gland, the hypothalamic-pituitary-gonadal axis lacks the stimulus for consecutive testosterone production and release in the periphery, ultimately leading to significantly decreased testosterone serum concentrations to castration levels within 2–4 weeks (Turner and Briken 2018). Antiandrogen use during the first week has been recommended by several authors to prevent increases in (paraphilic) sexual behaviours in relationship with a ‘flare up’ effect (Thibaut et al. 1993; Turner and Briken 2018). GnRH agonists do not interfere with the actions of androgens of adrenal origin.

Single case reports of successful GnRH analogue treatment in patients with exhibitionism, CSB and neurological disorders have been published (Rich and Ovsiew 1994; Ott 1995). Three systematic reviews concluded, based on the current state of research, that GnRH agonists lead to greater reductions in sexual functioning and greater decreases in sexual fantasies and behaviours compared to SSRIs or CPA in patients with paraphilic disorders (Briken et al. 2003; Lewis et al. 2017; Turner and Briken 2018). The same seems to account for CSBD, however, studies are scarce (Landgren et al. 2022). In contrast to CPA, however, treatment with GnRH agonists leads to complete suppression of paraphilic as well as non-paraphilic sexuality in most patients, which on the one hand may be a desirable effect, but on the other hand may also significantly limit their use, as a complete suppression of sexuality may be the primary therapeutic goal in only a very small proportion of treated patients with severe paraphilic disorders (Basdekis-Jozsa et al. 2013; Turner et al. 2019).

The adverse effect profile of GnRH agonists is comparable to that for CPA, although the described adverse effects seem to occur somewhat less frequently than under treatment with CPA, as detailed in (Thibaut et al. 2020). However, special consideration should be given to the regularly observed decrease in bone mineral density often seen with prolonged use of GnRH agonists, which may only partially return to normal after discontinuation of the medication (Turner and Briken 2018). Therefore, bone mineral density measurements should be done regularly during treatment with GnRH agonists, and appropriate counteractive treatment should be initiated if decreases in bone mineral density exceeds a certain threshold (Thibaut et al. 2020). Furthermore, with prolonged treatment, an unfavourable effect on various cardiovascular factors has been observed in older men with prostate carcinoma. Weight gain, hypertension, increased insulin insensitivity, hyperlipidaemia, and increasingly fibrotic remodelling of penile and testicular tissues, which may be associated with pain, permanent sexual dysfunction, and permanent infertility, have been reported (Sciarra et al. 2016).

### Empirical findings concerning pharmacological treatment of paraphilic disorders with relevance for the treatment of CSBD

Most patients with CSBD have conventional sexual fantasies, impulses, and behaviours and need better control over them. In patients with paraphilic disorders, patients have unusual and problematic fantasies and behaviours. The current WFSBP guidelines on the pharmacological treatment of adult paraphilic disorders recommend three different agents: SSRIs, antiandrogens (namely CPA), and GnRH agonists (Thibaut et al. 2020). The current available data (one randomised short-term controlled study) on the use of GnRH antagonists in paraphilic disorders is insufficient to recommend them at this stage (Landgren et al. 2020). Interestingly, after 10 weeks of treatment, a significant reduction in CSB occurred compared to placebo treatment (Landgren et al. 2020,2021). Moreover, Sciarra and colleagues’ meta-analysis conducted in 2016 showed a similar good profile for GnRH agonists and degarelix (GnRH antagonist), with a low rate of discontinuation due to adverse events. The only significant difference was related to injection-site reactions rate that were significantly higher with degarelix. Finally, only CPA and 3-month triptorelin have obtained marketing authorisation in Europe for the indication of severe paraphilic disorders.

In conclusion, in clinical practice (particularly in forensic populations), an assessment of criminal history and paraphilic interests in CSB individuals and vice versa should be systematic in order to better target patients’ needs (especially criminogenic ones). The WFSBP guidelines have highlighted preferred combination of psychotherapy and antiandrogens in the case of adult paraphilic patients at high risk of sexual acting-out such as those with paedophilic tendencies or who have committed rape with sexual sadism, especially when CSB is observed (Thibaut et al. 2020). The current WFSBP guidelines concerning the pharmacological treatment of paraphilic disorders define four treatment aims (Thibaut et al. 2020):

- Control of paraphilic fantasies and behaviours in order to decrease the risk of a sexual offence,
- Control paraphilic sexual urges,
- Decrease the level of distress of persons with paraphilic disorders,
- Enhance non-paraphilic sexual interests and behaviours.

Thereby, the current state of literature indicates that by simply using any kind of medication the content of the paraphilic disorder is seldom changed (Turner and Briken 2018). The currently recommended pharmacological agents rather aim at increasing control over sexual (paraphilic) fantasies, behaviours, and urges.

### Non-invasive brain stimulation

Malandain et al. (2020) reported the first open-label case report of a positive effect of transcranial direct current stimulation (tDCS) in a patient who was reporting severe chemsex behaviour associated with CSB and addiction to several illicit drugs used in sexual contexts. CSB was decreased and concomitant illicit drug use was stopped, resulting in the disappearance of chemsex behaviour. This positive effect occurred after 5 days of daily sessions of right dorsolateral prefrontal cortex (DLPFC) stimulation and did not return after 8 months of follow-up. Similarly, in previous studies, tDCS has been used to decrease craving and/or use in tobacco- and alcohol-use disorders. In most cases, the DLPFC was targeted, and the anode was placed over the right DLPFC (Lefaucheur et al. 2017). These findings also resonate with tDCS findings in improving regulation of gaming urges and emotions in individuals with internet gaming disorder (Wu, Zhu, et al. 2020; Wu, Potenza, et al. 2020; Wu et al. 2021). The increase in DLPFC activity may have modulated limbic pathways and reduced incentive salience and craving of both sexual behaviour and concomitant illicit drug use. However, further studies are needed to confirm this promising result.

### Chemsex use associated with CSB or CSBD

Chemsex practice does not necessarily mean harm to an individual’s sexual or psychological health. Chemsex use may be associated with CSB or CSBD and/or addiction to illicit drugs that affect individuals’ daily lives or their physical and/or mental health. Most individuals engaging in chemsex do not self-identify with problematic drug use and often report no negative consequences in everyday life. However, the frequent association of chemsex with lower life and sexual satisfaction suggests that psychosocial support or treatment may be needed for some individuals engaging in chemsex (Hibbert et al. 2019). Chemsex is not a psychiatric disorder and may be considered a syndromal description. As there are no treatment guidelines for chemsex so far, we briefly present the possibilities of pharmacological interventions that may be considered in cases of co-occurring CSBD and chemsex.

Promoting harm reduction, enhancing education, especially regarding infectious dieases or drug toxicity, are important for people engaging in chemsex (e.g., teaching about condom use or illicit drug use associated risks, regular monitoring of serologies, pre-exposure prophylaxis (PrEP) and post-exposure prophylaxis (PEP) use, etc.). In several countries, supportive health, and community sector interventions have been implemented in order to improve the wellbeing of people engaging in chemsex. Interestingly, Sewell et al. (2019) reported a decline in the prevalence of chemsex (31.8–11.1%) during a 6-month follow-up in 622 MSM, of whom 400 were still engaged with the study at the end of the follow-up. Mephedrone and GHB/GBL use significantly declined and crystal methamphetamine use did not change. They concluded that health promotion was beneficial in MSM with problematic chemsex.

In cases of alcohol-use disorders, the related WFSBP guidelines may apply (Soyka et al. 2017). In cases of illicit-drug-use disorders, to date, no pharmacological treatment has been approved for methamphetamine, GHB/GBL or cathinone addictions. Moreover, there are no guidelines for effective management of disorders involving use of metamphetamine, ketamine, synthetic cathinones or GHB/GBL. Yet, Reback and Fletcher (2014) reported that MSM were able to reduce sexual risk behaviours and that participants were able to maintain these reductions after treatment for the abuse of illicit drugs (especially methamphetamine). Thereby, they could reduce high-risk sexual behaviours (Reback and Fletcher 2014).

The misuse of GHB/GBL during chemsex may be associated with an increase in intoxication, dependence and withdrawal, but the prevalence of GHB/GBL misuse disorders remains low. In case of GHB/GBL dependence, detoxification is challenging, and hospitalisation is recommended in cases of severe dependence. Kamal et al. (2017) have reviewed GHB dependence and withdrawal symptoms, which are close to those reported with alcohol withdrawal and may include life-threatening symptoms. They have proposed an algorithm for the treatment of GHB withdrawal using benzodiazepines. The GABA_B_ receptor agonist baclofen may be of interest with respect to maintaining abstinence in these patients. In an open study, Beurmanjer et al. (2018) reported the efficacy of baclofen (45–60 mg/day) in 37 GHB-dependent patients as compared to treatment as usual in 70 GHB-dependent patients. Baclofen was associated with reduced relapse and drop-out rates.

No treatment guidelines exist for the management of ketamine intoxication or withdrawal symptoms; there are only case series and case reports (Lim 2003). Garg et al. (2014) have reported a case of naltrexone efficacy in the treatment of ketamine dependence.

Imipramine, sertraline, buproprion and methylphenidate have been studied in randomised clinical trials in the treatment of methamphetamine-use disorder. These treatments have failed to show any substantial effects on methamphetamine use or craving (Galloway et al. 1996; Shoptaw et al. 2006; Anderson et al. 2015). However, most studies were conducted in MSM with or without HIV infection. In many studies, the primary outcome was harm reduction or HIV infection prevention. McElhiney et al. (2009) reported a positive effect of the combination of CBT and modafinil. Among the ten HIV-infected MSM who completed treatment, six reduced their crystal methamphetamine use by more than 50%. However, there was no control group, and the positive effect might be due to modafinil, CBT, both or neither. In a recent review, Siefried et al. (2020) concluded that while no pharmacological treatment demonstrated convincing results, some positive findings have been reported with stimulants (dexamphetamine and methylphenidate), naltrexone and topiramate. Less consistent effects have been shown with bupropion and riluzole (a glutamatergic agent). Mirtazapine (30 mg/day) reduced the use of methamphetamine and risky sexual behaviours at week 24 of treatment, and the effect was maintained 12 weeks after treatment in 150 MSM practicing chemsex and using methamphetamine (Coffin et al. 2020). Other antidepressant medications (SSRIs, tricyclic drugs) have not been effective in reducing chemsex use. In another review, Lam et al. (2019) concluded that there was insufficient evidence to support the use of naltrexone in metamphetamine-use disorder. Finally, varenicline (1 mg BID) was not effective for metamphetamine-use disorder (Briones et al. 2018).

Lev-Ran (2012) described the case of a young man with cathinone dependence and depression who was treated with bupropion. Bupropion is a synthetic cathinone with a dual dopamine-norepinephrine reuptake inhibiting mechanism. It was approved in several countries for the treatment of depression and/or smoking cessation. In real-world settings, misuse of bupropion may occur but is quite uncommon (Naglich et al. 2019).

Phosphodiesterase-5 inhibitors (PDE5i) (sildenafil, vardenafil, tadalafil, avanafil) on demand or daily are an efficient symptomatic treatment in patients with erectile dysfunction. Intracavernous injections of prostaglandin E1 or vacuum pump treatments may constitute second-line treatments (for review see Füllhase et al. 2014; Yafi et al. 2018), about their tolerance). Erectile dysfunction is frequently observed in people engaging in chemsex. These individuals should be aware of the cardiovascular risks and an increased risk of death associated with the combination of PDE5i and illicit drugs (especially GHB/GBL, methamphetamines and cathinones).

## Guidelines

### Evaluation of patients

The first step is to evaluate the patient. Comparable to other mental disorders, a detailed biographical, psychiatric, somatomedical and addiction-specific history should be taken during the diagnostic process. Special emphasis should be placed on taking a structured sexual history and on the assessment of co-occurring psychiatric – including paraphilic – disorders because these could influence the choice of pharmacological intervention.

For many patients, their sexual behaviour may be sensitive, so sufficient time should be planned for taking a sexual history (Turner, Schöttle, et al. 2014). Taking a sexual history may extend over several appointments, which may give patients time to overcome initial inhibitions and open up. Especially at the beginning of the diagnostic process, a patient’s representations of his or her own sexuality may be distorted by socially desirable response styles. It is also not uncommon for patients to initially seek medical or psychotherapeutic help at the urging of their partner, family, supervisor, or court, so this may also lead to distortions in patients’ accounts. Obtaining a history from others, for example from the partner or other close individuals, as well as requesting prior medical/psychotherapeutic records may help resolve possible discrepancies in a patient’s presentation and diagnostic process.

A structured biographical and sexual history should include at least the following points:

- Age, gender, education, occupational status
- History of uro-genital, endocrinological, neurological or other somatic disorders
- Family and personal history of psychiatric and/or addictive disorders (with or without substances)
- Personal history of suicide attempts
- Personal history of brain trauma
- Current dementia, mental retardation
- Previous and current pharmacological treatments for psychiatric and somatic diseases
- Age of puberty
- Sexual orientation
- Previous sexual experiences
- Past history of sexual abuse and other traumas
- Early exposure to pornography
- Current sexual behaviour, type and frequency of various sexual behaviours (masturbation, intercourse, use of internet pornography, etc.)
- No, changing, or steady sexual partners
- Sexual functioning, sexual pain
- Sexual fantasies, masturbation fantasies, sexual likes/dislikes, including paraphilic sexual preferences (i.e., paraphilic fantasies and activity, type and number of paraphilic disorders if any)
- Chemsex practices: alcohol or illcit drug use before or during sexual activities
- Cognitive distortions about sexuality, empathy, coping with stress, impulsivity, interpersonal relationships, insight, motivation for treatment, denial
- Forensically relevant sexual behaviour
- Past or current history of STIs (HIV, hepatitis, syphilis, others)
- Use of sexual violence or constraint during sexual relationships, risky sexual behaviours
- History of financial difficulties related to sexual behaviours
- Previous treatments for CSBD or other sexual problems, adverse effects and compliance
- Current motivation for treatment

With information gleaned from such an assessment, a general overview of a patient’s biography, sexual development, and current sexual life may be obtained without already drawing conclusions about a possibly existing disorder.

To quantify the number of sexual behaviours and fantasies and to decide whether they reach a level that could possibly be considered as pathological, one may make use of structured assessment instruments described in section ‘Assessment of CSBD’. These instruments can assist with the diagnostic process; however, a diagnosis should not be solely based on the results of these instruments. Furthermore, the answers to single items could provide the basis for further discussions during the diagnostic and therapeutic process, especially in cases of ambiguity. For a final diagnosis of a CSBD, it is important to consider the significance of any personal suffering or interpersonal problems related to CSB. According to Rosenberg et al. (Rosenberg et al. 2014), the following questions may be helpful. Additionally, we added some further questions:

- How strong has the intensity of sexual desire throughout life been? How pronounced are the sexual self-regulation abilities?
- What role do differences in sexual desire between partners play when a partnership exists? Did the partner initiate the appointment?
- What role do moral and religious attitudes play (for example towards pornography or extramarital sexual contacts)?
- What is the function of CSB for the patient?
- Are positive (e.g., stimulation) or negative reinforcement mechanisms (e.g., coping with stress, anxiety and depression) meaningful?
- What roles do substance-related disorders and other mental disorders play?
- What roles do sexual risk behaviours and STIs play?
- What roles do risks of pregnancy in women, non-consensual sexual behaviours, or dependence or intoxication with illicit drugs or alcohol play?

The diagnostic process should be completed with an orienting physical examination, including a neurological examination and examination of the primary sex organs. If there are reasonable grounds for suspicion, the diagnostic process may be supplemented with laboratory tests, including determination of serum hormone concentrations or further organ-specific diagnostics (e.g., sonography, neuroimaging, genetic testing). HIV testing, hepatitis serology determinations, and testing for STIs should be conducted as indicated. Standardised methods of assessment, including assessment tools, may help to facilitate treatment strategies. Such methods could include the assessment of intellectual and personality functioning or psychopathology and the assessment of sexual behaviour and minimisation or denial of CSBD.

At the end of the diagnostic process, a biopsychosocial model may be developed together with the patient and a diagnosis of a CSBD based on the ICD-11 should be made or ruled out. Co-occurring paraphilic disorders and chemsex practice should be assessed and considered as they may influence therapeutic decisions. One should consider that the current ICD-11 diagnostic criteria state that in cases in which psychological distress due to CSB is entirely related by moral-religious beliefs, that this is insufficient to make a diagnosis of CSBD. In cases in which a diagnosis of CSBD is made, the patient should be informed about possible/indicated treatment options. These should be discussed together with the patient in order to promote shared therapeutic decision-making. Finally, motivational interviewing is not mentioned in the published studies, but poor motivation is often a significant factor relating to non-compliance, and motivation for treatment may be assessed and targeted in treatment as appropriate (Ball et al. 2006; Andersson et al. 2018).

### Psychotherapy

First-line treatment should include psychotherapeutic interventions. In some patients, psychoeducational interventions may be sufficient to reduce CSB and related psychological distress (Hardy et al. 2010; Hall et al. 2020). Psychoeducation has the advantage of being a low-cost intervention that may be easily disseminated to a variety of patients. For example, psychoeducational programs available online may reach people living in rural regions where specialised treatment providers may be distant or not available. However, psychoeducation has limits. Psychoeducation is often not individualised, and it typically lacks direct interactions between patients and treatment providers. On the other hand, since patients initially may feel uncomfortable when talking about their sexuality, psychoeducation could be a first step to reduce barriers within psychotherapy. Thus, psychoeducation may be an easy-to-perform entry into psychotherapeutic processes in face-to-face psychotherapeutic interventions. Psychoeducation may include a risk-reduction program adapted to CSBD (i.e., reducing the risk of STIs and unwanted pregnancies), and in cases involving chemsex, education about the risks associated to illicit drug use may be included.

In patients in whom psychoeducation is insufficient to relieve psychological distress or reduce symptoms of CSB, psychotherapeutic interventions should be used. So far, CBT and ACT in single and group settings have shown positive effects concerning the reduction of impairment and distress related to CSB. Thereby, psychotherapy should be provided by a psychologist or medical health care provider, best with additional training in sexual medicine. Within the psychotherapeutic process, co-occurring psychiatric/addictive/paraphilic disorders should be assessed and addressed as appropriate, and pharmacological treatment may be necessary (please refer to specific WFSBP guidelines on these topics). As described in section ‘Psychological treatment of CSB,’ there exist evaluated psychotherapeutic treatment programs specifically designed for people suffering from CSB (Garcia et al. 2016; Hallberg et al. 2019).

### Pharmacological treatment

In some patients, sexual fantasies, urges or behaviours may be so intense that psychotherapy alone is not sufficient to reduce psychological distress. Especially at the beginning of the therapeutic process, CSBs may be so overwhelming and irresistible that the patient is not capable to transfer the learned strategies into his/her daily life. In these patients, additional pharmacological treatment may be helpful or even necessary.

Pharmacotherapy can have the following aims:

- Reduce compulsive-sexual fantasies, urges or behaviours;
- Enhance self-control over compulsive-sexual fantasies, urges or behaviours;
- Treatment of co-occurring psychiatric/addictive or somatic disorders associated with compulsive-sexual fantasies, urges or behaviours;
- In case of chemsex, control chemsex behaviour and use of illicit drugs;
- In case of co-occurring paraphilic disorders, to reduce the risk of sexual offending.

Being neglected or sexually abused as a child are significant factors that may be associated with CSBD. Specific psychological treatment of physical or sexual trauma is an essential component of treatment. Sexual abuse treatments are available in many countries, and professional addresses are available on the internet.

#### Recommended pharmacological agents

##### SSRIs

SSRIs may be indicated, especially for patients in whom CSBs present themselves phenomenologically rather as obsessive-compulsive behaviours. Patients with a more obsessive-compulsive pattern of CSB may experience particularly intrusive sexual fantasies, and the sexual urges may generate feelings of anxiety, disgust or unease, which may be relieved through acting out the intrusive sexual fantasies. Prevalent co-occurring psychiatric disorders in patients with a CSBD are depressive and anxiety disorders. As SSRIs are first-line pharmacological treatments for depressive and anxiety disorders, SSRIs may be especially suitable for patients with an additional depressive or anxiety disorder. Furthermore, by increasing the level of serotonin, SSRIs may decrease impulsivity and libidinal drive.

So far, no differences between SSRIs have been found concerning their efficacies to reduce CSBs; however, most studies have been conducted with fluoxetine and sertraline and there is one recent RCT that has successfully evaluated the use of paroxetine. Furthermore, some investigators propose that dosages should be comparable to those usually prescribed for the treatment of OCD (e.g., at the higher end of the range of sertraline 50–200 mg/d or fluoxetine 20–80 mg/d). In case one aims for a reduction of sexual desire and sexual arousal, it was found that citalopram, paroxetine, fluoxetine, sertraline and venlafaxine are SSRIs with high rates of patients with sexual desire or arousal dysfunctions (Serretti and Chiesa 2009). However, one should keep in mind that especially in men, SSRI treatment may also be associated with erectile dysfunctioning, delayed ejaculation and inhibited orgasm which may increase non-compliance to treatment. Problems with erectile functioning can even persist after SSRI treatment has been terminated (Balon 2006; Bala et al. 2018).

**Conclusions and recommendations:** Though not formally approved for CSBD, SSRIs have been included in clinical practice ‘off label’ for the treatment of CSBD, although more research demonstrating efficacy is needed. Despite only two randomised controlled studies, there is some further clinical evidence that all SSRIs reduce CSB with a reasonable benefit/risk ratio (level B of evidence). SSRIs may be especially suitable for patients with comorbid depressive, anxiety or obsessive-compulsive disorders.

Before and during SSRI treatment, the following routine examinations should be performed at least every three to 6 months (in patients above 60 years of age in any case every 3 months) and every year for biological measurements (blood sugar and lipid profile) and electrocardiogram.

- Exclusion of contraindications or interactions with other medications. Caution in case of past history of suicidal attempts.
- Electrocardiogram (especially QT_c_ interval), blood pressure, pulse
- Obtaining blood samples including complete blood count, creatinine, electrolytes, liver enzymes, lipid profiles, and fasting blood glycaemia (HbA_1C_ if necessary)_,_
- Body weight, BMI

##### Naltrexone

Naltrexone may be indicated in patients in whom CSBs reflect an addictive behavioural pattern showing symptoms such as craving, tolerance and withdrawal. In these patients, CSBs may be experienced as ego-syntonic. As described in the previous sections, there exist some studies suggesting naltrexone to be effective at doses ranging from 50 to 150 mg/d in reducing CSBs. As naltrexone is frequently used in patients with SUDs (where it is indicated for alcohol and opioid use disorders), it seems to be especially suitable for patients with CSBD with co-occurring SUDs. The same may hold for patients with co-occurring behavioural addictions, like gambling or gaming disorders. The tolerability and efficacy of naltrexone was confirmed in a placebo controlled, randomised controlled trial. Furthermore, in several case reports, naltrexone has shown good efficacy as an add-on to SSRIs which were previously not efficient (e.g., Grant and Kim 2002; Bostwick and Bucci 2008). Naltrexone in conjunction with SSRIs may also be helpful in cases of co-occurring CSBD and mild paraphilic fantasies (personal communication 2022, Thibaut; unreferenced).

**Conclusions and recommendations:** Though not formally indicated for CSBD, naltrexone has already been included in clinical practice ‘off label’ for the treatment of CSBD, although more research demonstrating efficacy is needed. Despite one RCT and few controlled studies, there is some clinical evidence that naltrexone may reduce CSB with a reasonable benefit/risk ratio (level B of evidence). Naltrexone may be especially suitable for patients with co-occurring addictive disorders (substance as well as behavioural addictions).

Before and during naltrexone treatment, the following routine examinations should be performed at least every three to 6 months (in patients above 60 years of age in any case every 3 months):

- Exclusion of contraindications; e.g., acute opioid use disorder with current opioid use or current opioid treatment, acute hepatitis or severe hepatic dysfunction, congenital galactosemia. Caution in case of past history of suicidal attempts.
- Drug screening before treatment, especially opioids.
- Obtaining blood samples, especially liver enzymes but also including complete blood count, creatinine, electrolytes.

##### Other drugs and other forms of treatment

Topiramate (50–200 mg/day) or nefazodone (50–400 mg/day) may be used off-label (no level of evidence), particularly in individuals with co-occurring depressive disorders. However, their use cannot be recommended on a regular basis due to a lack of research. Stimulants may be used in patients with CSB and co-occurring ADHD (Kafka and Hennen 2000), although prescribers should be aware that people with CSB may use stimulants during chemsex. A first case series suggests that NAC (up to 3600 mg/d) might be useful in patients with CSBD as well, however, clearly more studies are needed. Non-invasive brain stimulation may also be considered (there is a positive report with tDCS stimulation of the right prefrontal cortex (Malandain et al. 2020; no level of evidence)).

**Conclusions and recommendations:** Though other psychotropic drugs such as topiramate, nefazodone or psychostimulants may be used in specific cases, the level of evidence for the use of these drugs is poor when there are no co-occurring psychiatric disorders (case reports, small sample sizes, lack of power, lack of controlled studies) (level E of evidence).

##### Cyproterone acetate (CPA) (see also Thibaut et al. 2020 WFSBP guidelines on the pharmacological treatment of paraphilic disorders)

CPA may be of interest in patients with CSBD and co-occurring paraphilic disorders. CPA may be given orally at doses between 50 and 200 mg/d or intramuscularly at dosages between 200–400 mg/d every two to four weeks. Compared to SSRIs or naltrexone, adverse drug reactions are found more frequently during treatment. These adverse drug reactions result primarily from the reduced testosterone action on receptors and range from rather harmless adverse effects, such as hot flashes, asthenia, or pain at the injection site, to more serious adverse effects, such as depression, gynaecomastia, thromboembolic events, or liver dysfunctions (Assumpcao et al. 2014). After discontinuation of the medication, most of the adverse effects are typically (completely) reversible. Of special importance, it has been found that CPA treatment is associated with the occurrence of meningiomas, whereby a dose-dependent effect has been reported (Gil et al. 2011). Thus, cMRT should be conducted before and at least once a year during CPA treatment. In case a meningioma develops, CPA treatment should be stopped as soon as possible. Due to these adverse effects, CPA treatment should be reserved for patients with intense CSBD symptoms and co-occurring paraphilic disorders at a moderate or greater risk of sexual offending. In several countries (such as France), a written informed consent renewed every year is mandatory, and CPA should not be used for CSBD unless there are no other therapeutic options (Turner et al. 2019).

**Conclusions and recommendations:** Though not formally approved for CSBD, CPA has been used in clinical practice for the treatment of CSBD in specific cases, although more research demonstrating efficacy is needed. Despite one uncontrolled study and one case report, there is some clinical evidence that CPA may reduce CSB (level C1 of evidence). However, due to the possibility of severe adverse effects, CPA treatment should be reserved for patients with intense CSBD symptoms and co-occurring paraphilic disorders with an at least moderate risk of sexual offending.

Before and during CPA treatment, the following routine examinations should be performed at least every three to 6 months (in patients above 60 years of age, every 3 months):

- Exclusion of contraindications such as severe hepatic dysfunction, meningioma, severe osteoporosis, past history of thromboembolism, tuberculosis, severe diabetes, puberty not completed, severe and chronic depression, congenital galactosemia
- Obtaining blood samples, especially calcium and phosphate levels and liver enzymes and also complete blood count, creatinine, electrolytes, lipid profiles, and fasting blood glucose levels (if necessary HbA1_c_)
- Plasma hormone levels, including free testosterone, LH, prolactin at baseline
- Body weight, BMI
- Osteodensitometry at least once a year in case of risk of osteoporosis or age >50; if not, every 2 years
- MRI brain scan at least once a year

#### GnRH agonists

GnRH agonists are only recommended in cases of patients with co-occurring CBSD and paraphilic disorders with high risks of sexual violence. The use of GnRH agonists usually leads to a complete decline of sexual functioning and should thus only be used in patients with severe CSBD symptomatology and severe paraphilic disorder with high risk of contact sexual offending (e.g., paedophilic or sexual sadism disorders). When using GnRH agonists, it should be noted that the desired effect only occurs two to four weeks after the start of treatment. In the first two to four weeks of treatment, GnRH agonists may lead to increased plasma testosterone levels (flare-up effect) and can thus even lead to increases in CSB. Therefore, the first two to four weeks of GnRH-agonist treatment should be accompanied by additional CPA treatment.

**Conclusions and recommendations:** Though not formally approved for CSBD, GnRH agonists have been used in clinical practice for the treatment of CSBD with specific features, although more research demonstrating efficacy is needed. Until now, there are no specific studies evaluating the use of GnRH agonists to reduce CSB (level C3 of evidence). Due to the possibility of severe adverse effects, GnRH-agonist treatment should be reserved for patients with intense CSBD symptoms and co-occurring paraphilic disorders with high risk of sexual offending.

Before and during GnRH-agonist treatment, the following routine examinations should be performed at least every 3–6 months (in patients above 60 years of age, every 3 month):

- Exclusion of contraindications such as osteoporosis
- Obtaining blood samples, especially calcium and phosphate levels and liver enzymes and complete blood count, creatinine, electrolytes, lipid profiles, and fasting blood glucose levels (if necessary HbA1_c_)
- Plasma hormone levels, including free testosterone, LH, prolactin
- Body weight, BMI
- Osteodensitometry at least once a year in cases of risk of osteoporosis or age >50

#### Medical monitoring is necessary during pharmacological treatment

In all cases, sexual activity and fantasies (nature, intensity and frequency) should be evaluated at baseline and at least every month through self-reports of the patient and, if useful and necessary, by interviewing family members or caregivers. Assessment scales or daily planners may be helpful to help patients measure their behaviours.

At baseline, weight/body mass index, blood pressure, electrocardiogram, renal and liver function, blood cell counts, fasting blood glucose levels and lipid profiles should be systematically assessed. Concerning the monitoring, guidelines may differ according to countries. Body weight, blood pressure, glucose and lipid profiles should be regularly checked with SSRI treatment. Liver function should be monitored in case of naltrexone treatment and electrocardiograms should be checked in cases of any cardiac symptoms with SSRIs and naltrexone. Caution is required when SSRIs are used in adolescents (they may increase the risk of suicide). Similarly, naltrexone could also increase the risk of suicide in all patients. In case of antiandrogen treatments, the WFSBP guidelines apply (see Table 1 in Thibaut et al. 2020).

### Guideline limitations

Most reports on the treatment of CSBD are case reports or series. In general, treatment efficacy studies are marked by methodological biases. The identification of standardised and reliable measures of sexual behaviour is difficult. Self-reports of sexual activity are usually used, but they do not constitute reliable indices of sexual behaviour. Reliable methods of assessment are lacking. Finally, controlled and randomised pharmacological studies are scarce, and studies are almost absent in women.

National or international collaborative studies, including large cohorts of well-defined CSBD with long durations of follow-up, are needed to confirm these preliminary data on the efficacy of some pharmacological treatments for CSBD.

Comparisons between studies are often difficult due to methodological differences including durations of follow-up, types of CSBD with heterogeneity within and across samples, retrospective or prospective studies, types of treatment and compliance, statistical analyses, and adverse effects or dropout rates often not being reported, among others.

In addition, specific problems may occur when randomisation is adapted to psychological treatments. Therapists may have significant impacts on therapeutic outcomes if they, for example, adapt treatments to the learning styles and interpersonal approaches of patients and adjust therapies to fluctuations in individuals’ motivations and moods. The controlled study design may not facilitate features of productive therapist-patient relationships.

## Treatment algorithm

Due to the limited number of resources and randomised controlled studies available, these guidelines are ‘pragmatic guidelines’ with less rigorous methodological standards than those used for most of WFSBP guidelines as described in Hasan et al. (2019). We encountered similar difficulties with the previous WFSBP guidelines on the treatment of paraphilic disorders in adults (Thibaut et al. 2020).

The aim of the treatment of CSBD using an integrated approach (Briken 2020; Briken and Turner 2021) is to find a satisfactory balance between excitatory and inhibitory factors related to sexual behaviour by doing the following, as indicated:

- Developing awareness of CSB/CSBD and motivation for change;
- Building a network of supportive people;
- Reducing shame associated with CSBD and restoring self-esteem;
- Teaching personal skills through communication;
- Addressing chemsex behaviour and treating co-occurring disorders (e.g., anxiety, depression, paraphilic disorder);
- Helping patients reacquire control of sexual behaviour and develop a healthier approach to sexuality;
- Decreasing the level of distress in persons with CSBD;
- Managing financial and legal consequences;
- Treating somatic consequences such as STIs.

Treatment choices may depend on the following:

- The patient’s previous medical, psychiatric, sexual and criminal history;
- The patient’s compliance and motivation for treatment;
- Co-occurring somatic and psychiatric disorders;
- The severity of CSBD;
- The type of CSBD (predominantly compulsive, impulsive or addictive type; autoerotic or partnered);
- The association of CSBD with paraphilic disorders or chemsex practices.

Psychiatric disorders such as ADHD may enhance the risk of developing CSB, which suggests that future research may be well-served by attempting to identify specific risk factors for the development of CSBD. Finally, there is evidence for the conclusion that CSBs often co-occur with a range of other impulsive and addictive behaviours such as substance use, problematic gaming, and problematic gambling, as well as with paraphilic disorders. Such comorbidity highlights the need to assess for CSBD in addiction treatment settings, particularly in settings where behavioural addictions may already be a focus of treatment, as well as in settings where patients with paraphilic disorders are treated. Because of the shame associated with CSB, patients often do not talk about it openly. Patients are often seen at the stage of psychiatric and/or somatic symptoms associated with CSB, and these may include suicidal ideation, depression, anxiety, addictive disorders, or infectious diseases. In many patients, histories of having experienced sexual abuse may require specific and appropriate care. Finally, some people engaging in chemsex may also fulfil criteria for CSBD.

In clinical practice, we will make a distinction between three main situations:

1. the patient has only CSBD without any chemsex practice and without any paraphilic disorder;
2. the patient has a co-occurring paraphilic disorder (mainly paedophilia, exhibitionism or sexual sadism);
3. the patient engages in chemsex and has CSBD. The patient may also have addictive disorders related to the use of illicit drugs during chemsex.

**(1) CSBD without chemsex practice and without paraphilic disorder** (Table 6)

**Table 6.** A proposed algorithm for pharmacological treatment of CSBD without any chemsex practice and without any paraphilic disorder, based on Briken and Turner (2021) and Thibaut et al. (2020).

Level of severity	Treatment
**LEVEL 1 – mild and mild to moderate**	
Aim: support the control of sexual fantasies, compulsions, and behaviours without risk of self-harm or harm to others	Psychoeducation (prevention of unwanted pregnancies, STIs; education about the risks associated with CSB) Motivational interviewing Psychotherapy, preferentially CBT or ACT (level C of evidence) Treatment of co-occurring depressive or anxiety disorders or addictive or other psychiatric disorders if any Reduce levels of stress and impulsivity and improve self-esteem
**LEVEL 2a – moderate**	
Aims: support the control of sexual fantasies, compulsions, and behaviours Specifiers: Presence of co-occurring depression or anxiety disorder No satisfactory results at level 1	Psychotherapy, preferentially CBT or ACT SSRI^a^: dosage gradually increased at the same level as prescribed in OCD (e.g., sertraline 50–200 mg/day or fluoxetine 20–80 mg/day or paroxetine 20–60 mg/day) (level B of evidence)
**LEVEL 2b – moderate**	
Aim: support the control of sexual fantasies, compulsions, and behaviours Specifiers: Presence of co-occurring alcohol or substance misuse, other addictive behaviours No satisfactory results at level 1	Psychotherapy, preferentially CBT or ACT Naltrexone^b^ 50–200 mg/day (level B of evidence)
**LEVEL 3 – severe**	
Aim: support the of control severe CSBD symptoms Specifiers: No satisfactory results at level 2a and 2b	Psychotherapy, preferentially CBT or ACT Add naltrexone^b^ (50–200 mg/day) to SSRI^a^ or SSRI^a^ to naltrexone^b^ (e.g. sertraline 50–200 mg/day or fluoxetine 20–80 mg/day or paroxetine 20–60 mg/day) (level C of evidence)

^a^Be careful in case of adolescents; there is an increased risk of suicide for SSRIs noted across age groups. National guidelines on antidepressant use monitoring may apply.

^b^Contraindications of naltrexone: acute hepatitis or severe hepatocellular insufficiency; concomitant use of opioids; pregnancy, lactation; suicidal risk; severe kidney failure; hypersensitivity to naltrexone or one of its components; individuals under 18 or over 65 years of age (French Health Authority 2015).

Though other psychotropic drugs such as topiramate, nefazodone or psychostimulants can be used in specific cases, the level of evidence for the use of these drugs is poor when there are no psychiatric comorbidities (Level E of evidence). The efficacy of non-invasive brain stimulation techniques needs further investigation.

CPA or GnRH agonist treatments should be reserved for patients with intense CSBD symptoms and co-occurring paraphilic disorders with an at least moderate risk of sexual offending.

Specific care in case of past history of sexual abuse is necessary.

**(2) CSBD with co-occurring paraphilic disorders (mainly paedophilia, exhibitionism, or sexual sadism)**

In patients with CSBD and co-occurring paraphilic disorders, the treatment algorithm of the guidelines for the treatment of paraphilic disorders should be used (see Thibaut et al. 2020).

**(3) CSBD associated with chemsex**

For this group of patients, the careful search for co-occurring psychiatric and somatic disorders and somatic and psychiatric consequences of alcohol and illicit drug use and chemsex practice is important. In all cases, psychoeducation is important (reducing the risk for STIs, alcohol and/or illicit drug intoxication and related adverse effects and non-consensual sexual behaviour and sexual violence; promoting use of rapid self-tests to search for STIs; informing about PrEP and PEP availability). Brief interventions for SUDs if any in addition to HIV prevention strategies should be discussed and used as indicated. In the case of alcohol use disorders, previous WFSBP guidelines may apply (Soyka et al. 2017). In case of illicit drug use disorders, to date, no pharmacological treatment is approved with indications for methamphetamine, cocaine, cannabis, GHB/GBL or cathinone addictions. No guidelines for effective management of methamphetamine, ketamine, synthetic cathinones or GHB/GBL use disorders exist presently.
